# Heparin-Tagged PLA-PEG Copolymer-Encapsulated Biochanin A-Loaded (Mg/Al) LDH Nanoparticles Recommended for Non-Thrombogenic and Anti-Proliferative Stent Coating

**DOI:** 10.3390/ijms22115433

**Published:** 2021-05-21

**Authors:** Shivakalyani Adepu, Hongrong Luo, Seeram Ramakrishna

**Affiliations:** 1 Center for Nanofibers and Nanotechnology, National University of Singapore, Singapore 119260, Singapore; shivakalyani@iith.ac.in; 2 Engineering Research Center in Biomaterials, Sichuan University, Chengdu 610064, China; hluo@scu.edu.cn

**Keywords:** bioachanin A, PLA-PEG-heparin, layered double hydroxide nanoparticles, controlled release, stent thrombosis, drug-eluting stent

## Abstract

Drug-eluting stents have been widely implanted to prevent neointimal hyperplasia associated with bare metal stents. Conventional polymers and anti-proliferative drugs suffer from stent thrombosis due to the non-selective nature of the drugs and hypersensitivity to polymer degradation products. Alternatively, various herbal anti-proliferative agents are sought, of which biochanin A (an isoflavone phytoestrogen) was known to have anti-proliferative and vasculoprotective action. PLA-PEG diblock copolymer was tagged with heparin, whose degradation releases heparin locally and prevents thrombosis. To get a controlled drug release, biochanin A was loaded in layered double hydroxide nanoparticles (LDH), which are further encapsulated in a heparin-tagged PLA-PEG copolymer. LDH nanoparticles are synthesized by a co-precipitation process; in situ as well as ex situ loading of biochanin A were done. PLA-PEG-heparin copolymer was synthesized by esterification reaction, and the drug-loaded nanoparticles are coated. The formulation was characterized by FTIR, XRD, DSC, DLS, and TEM. In vitro drug release studies, protein adhesion, wettability, hemocompatibility, and degradation studies were performed. The drug release was modeled by mathematical models to further emphasize the mechanism of drug release. The developed drug-eluting stent coating is non-thrombogenic, and it offers close to zero-order release for 40 days, with complete polymer degradation in 14 weeks.

## 1. Introduction

The implantation of a balloon expandable stent by percutaneous coronary angioplasty is the most commonly used medical intervention to re-open the occluded vessels. Earlier stents were mostly made of either Stainless Steel (SS) or Cobalt Chromium (CoCr) and were called Bare Metal Stents (BMS). A common problem associated with BMS implantation is in-stent restenosis, which occurs due to stent thrombosis, inflammation, and the proliferation and migration of Vascular Smooth Muscle Cell (VSMC) in the luminal area, which are collectively termed as neointimal hyperplasia (NH) [1,2,3].

Drug-eluting stents (DES) were developed in order to combat this problem, as the anti-proliferative drug coated onto the stent prevents NH. Nevertheless, late stent thrombosis (ST) emerged with the use of DES due to the impaired re-endothelialization owing to the non-selectivity of anti-proliferative drug and polymer hypersensitivity [4,5]. The conventionally used anti-proliferative drugs, such as paclitaxel, sirolimus, rapamycin, and its derivatives, tend to impede endothelial cell (EC) proliferation in addition to VSMC due to induced autophagy [6]. Dual drug-eluting stents comprising of anti-proliferative and endothelium-stimulating drugs have no benefit [7,8,9]. The re-endothelialization of luminal stent surfaces is of utmost importance, because the functional and complete endothelial cell lining prevents the adhesion, aggregation, and activation of blood platelets and thereby inhibits late stent thrombosis [10].

The selective nature of anti-proliferative drug toward VSMC and the hemocompatibility of the polymers used and for stent coating is vital [11]. As a result, further research is needed on newer drugs, newer polymers, and methods for stent surface modification to selectively inhibit intimal hyperplasia and stent thrombosis. Alternative drugs that have been employed in DES include probucol [12], carvedilol [13], succinobucol (AGI1067) [14], NADPH-oxidase inhibitors [15], superoxide dismutase inhibitors [16], anti-CD34 antibodies [17], resveratrol [18], quercetin [19], VEGF Gene [20], NO (Nitric Oxide) donors [21], which were reported to promote the endothelialization to an extent [22]. Various natural vasculoprotective agents that inhibit intimal proliferation and promote endothelial regeneration are sought, of which estrogens or estrogen receptor agonists are found to have excellent vasculoprotective activity [23,24]. There are two kinds of estrogen receptors: α receptor activation promotes uterorophic activity, whereas β receptor activation promotes vasculoprotective activity. Selective estrogen-β-receptor agonists are desirable to avoid uterotrophic activity. 17-β estradiol has been coated on DES; however, it is mostly unstable and difficult to extract [25,26]. Biochanin A is an isoflavone phytoestrogen and a selective estrogenic receptor β agonist that inhibits vascular cell adhesion molecule (VCAM) 1 upregulation. It has anti-proliferative, anti-migratory, anti-inflammatory, anti-oxidant, and vasculoprotective activities without uterotrophic activity. Thus, it can inhibit VSMC proliferation and migration without delaying re-endothelialization [27,28,29].

For effective inhibition of neointimal growth, the ideal polymer for DES coating should be non-thrombogenic, non-inflammatory, and non-toxic to cells, and it should encourage arterial healing by re-endothelialization. The stent surface should be hemocompatible to avoid thrombo-embolic processes until the re-endothelialization process is finished [30,31,32].

Various natural and synthetic non-biodegradable (poly-n-butyl methacrylate and polyethylene–vinyl acetate copolymer) and biodegradable polymers (polylactic acid, polylactic-co-glycolic acid, polycaprolactone, poly(lactide-co-σ-caprolactone), polyhydroxybutyrate, polyhydroxybutyrate-co-valerate, polyvinyl alcohol, phosphorile choline and chondroitin sulfate/gelatin) have been used for the delivery of anti-proliferative drugs on stent coating [33,34].

Non-biodegradable polymer coatings may raise the incidence of adverse cardiac events. Biodegradable polymers [35] and polymer-free DES [36] have been developed to reduce the polymer-associated inflammatory responses. Yet, the degradation products of biodegradable polymers may increase the occurrence of inflammatory reactions and subsequently cause NH. Fully degradable stents have been facing challenges in making a stent that has sufficient radial strength for an appropriate duration. Thicker struts provoke inflammatory response to a greater extent [37]. For polymer-free DES, in addition to the adverse interfacial inflammatory reactions, it is difficult to control the release kinetics of anti-proliferative drugs [36].

Even though a lot of research is ongoing, the perfect DES has yet to be developed. In addition to the prevention of restenosis, the following major tasks are being attempted: improvement of the vascular healing processes; antithrombotic coatings that remove the need for prolonged dual antiplatelet therapy; further development of controlled release polymer systems; tailored treatment with drugs that are multifunctional [22]. Non-thrombogenic surface coatings such as poly-l-lysine [38], dopamine [39], heparin [38], and quinone-rich polydopamine [40] have been reported to effectively inhibit ST yet suffer from the uncontrolled drug release which necessitates the need to tune the drug release profile.

Conventional biodegradable polymers used for drug-loaded coatings results in inflammation due to high local acidity throughout the degradation, which further accounts for thrombosis [41,42]. Hence, to develop a non-thrombogenic polymer system for drug release, heparin-tagged PLA-PEG (poly lactic acid–co-poly ethylene glycol) copolymer has been chosen, where PLA is a highly hydrophobic, biodegradable polymer and PEG has the ability to suppress cellular and protein adsorption and heparin suppresses the thrombosis [43,44]. Much research has been focused on the modification of PLA covalently with PEG, which includes the diblock polymer of PEG-PLA or a triblock polymer of PLA-PEG-PLA, using PEG of a molecule weight higher than 2KDa. PLA of high molecular weight with sufficient mechanical properties takes a longer time to degrade in the body, which can evoke immunological responses [45]. This copolymer has been chosen to tailor the degradation time as well as the hydrophilic properties of the polymer and to achieve controlled release of the drug. Heparin is a glycosaminoglycan and exerts its anti-coagulant activity by binding to anti thrombin III. It can be either covalently attached to the polymer or adsorbed on to the stent surface, which provides non-thrombogenic surface. As the result of the presence of many active functional groups in heparin such as −COOH, −OH, and −NH_2_, several chemical attachment options are available that allow bonding with a polymer backbone [46].

To acquire better stability and loading efficiency for biochanin A, the drug was loaded first in nanoparticles and later encapsulated in the polymer. Various inorganic nanoparticles have been used for drug delivery, including mesoporous silica nanoparticles, fullerenes, iron oxide nanoparticles, carbon nanotubes, and layered double hydroxides (LDH) nanoparticles [47,48]. Among which, LDH nanoparticles have been extensively investigated for drug delivery owing to the physicochemical stability, swellability, memory effect, high surface area, and high anion exchange capability [49]. LDH nanoparticles have been utilized widely for the delivery of anti-cancer drugs, therapeutic proteins, and genes [50,51]. It has not been reported earlier as a drug delivery carrier for stent coating application.

For the purpose, LDH nanoparticles also called anionic clays have been chosen; the mineral of this family is hydrotalcite (Mg-Al-CO_3_). The chemical composition of LDH is generally expressed as M (II) 1−x M (III) × (OH) 2 (An^−^) x/n × yH_2_O, where M (II) = divalent cation, M (III) = trivalent cation, A = interlayer anion, n- = charge on interlayer ion, and x and y are fraction constants. Inorganic or organic anions can be easily introduced between hydroxide layers by ion exchange or precipitation, as LDH is positively charged. A wide range of inorganic or organic anionic and cationic molecules can be replaced at the interlayers. Then, a neutral hybrid can enter cells by moving across a negatively charged cell membrane without repulsive electrostatic interactions that would be experienced by the guest anion alone [52]. Drug-loaded LDH nanoparticles are internalized into cells by phagocytosis or endocytosis. Once internalized into the cell, LDH is broken down by lysosomes and the release of drug takes place. The LDH, as a new drug carrier, is much simpler to synthesize in the laboratory; it has a high drug transportation efficiency, high drug-loading density, low toxicity to target cells or organs, and it also provides excellent protection to loaded molecules from undesired enzymatic degradation. Furthermore, its high chemical versatility and anionic exchange capacity are leading to a promising future in drug delivery and release [53]. LDH nanoparticles as such are challenging to coat onto the stent surface; thus, to attain a flexible and uniform coating as well as to offer a non-thrombogenic surface and further control the release, drug-loaded LDH nanoparticles are encapsulated in PLA-PEG-heparin copolymer.

Hence, in the present work, anionic drug biochanin A was loaded in Mg/Al (LDH) nanoparticles and further encapsulated with heparin-tagged PLA-PEG copolymer for stent coating.

## 2. Results

### 2.1. FTIR

**Figure 1:**
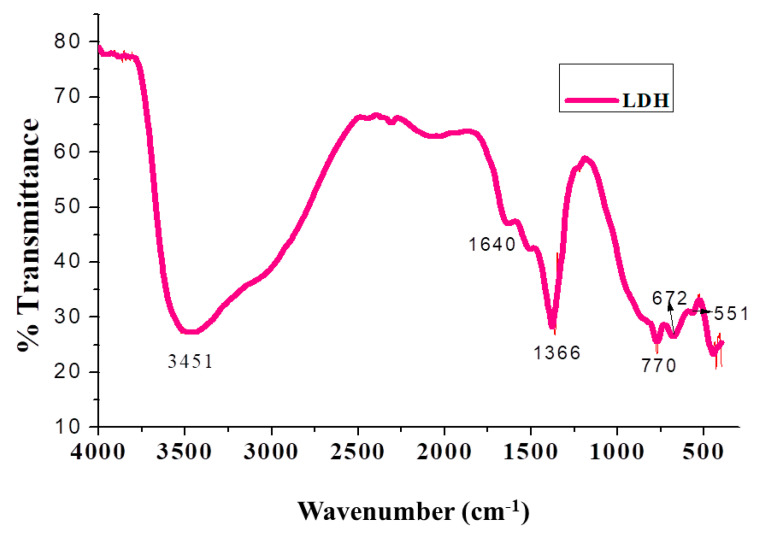
FTIR spectrum of Mg/Al-LDH.

The FTIR spectrum of (Mg/Al) LDH is represented in Figure 1, which exhibits a broadband at 3451 cm^−1^ attributed to hydrogen bonding of the interlayer water with interlayer CO_3_^−2^ anions. The band at 1640 cm^−1^ is due to the bending vibration of interlayer water. The antisymmetric *V*_3_ vibration of the CO_3_^−2^ band appears around at 1366 cm^−1^, and the peak at around 770 cm^−1^ is attributed to overlapping of the antisymmetric *v*_4_ vibration of CO_3_^−2^ and M-O stretching vibration. The bands at 672 and 551 cm^−1^ are associated with the translation mode of M-OH. The synthesized LDH nanoparticles are devoid of impurities, and all the peaks are consistent with the reports [54].

**Figure 2:**
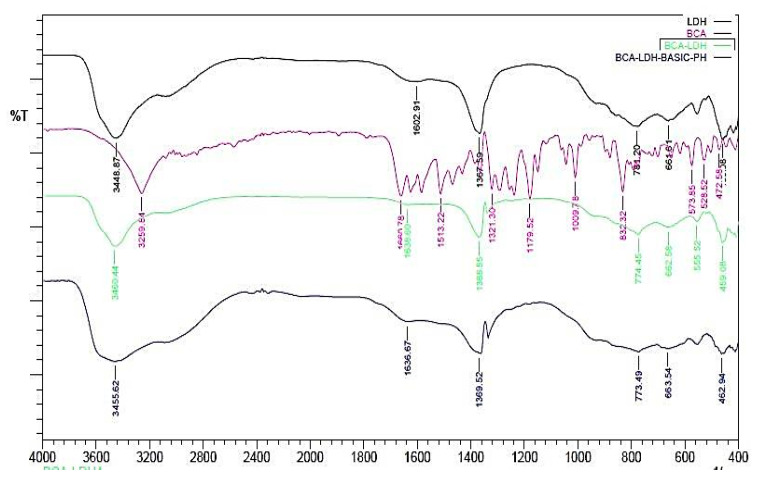
Overlay of FTIR of BCA-LDH-I, BCA, and LDH.

The FTIR of BCA and BCA-LDH-I is shown in Figure 2. The peak at around 1620cm^−1^ corresponds to C=O stretching, which confirms the bond between the methoxy group of BCA and the OH^−1^ of the interlayer water molecules of LDH, confirming the intercalation of BCA in LDH. The remaining peaks are similar to LDH [55].

**Figure 3:**
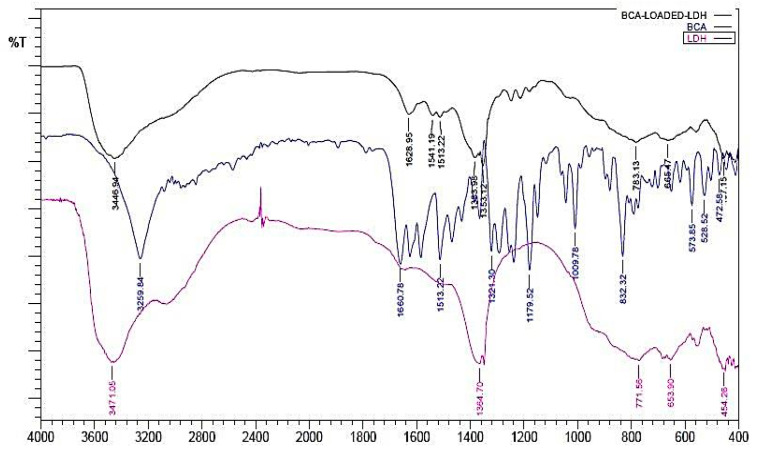
Overlay of FTIR of BCA-LDH-C, BCA, and LDH.

FTIR of BCA-LDH-C is represented in Figure 3. It consists of peaks corresponding to LDH as well as BCA. The broad peak at 3446 cm^−1^ corresponds to the OH^−1^ peak of LDH. The peak at around 1600 cm^−1^ corresponds to C=O stretching, which confirms the bond between the methoxy group of BCA and the OH^−1^ of the interlayer water molecules. The peak at around 1360 cm^−1^ corresponds to the –C-H stretching of BCA. The peak at around 600 cm^−1^ corresponds to M-O stretching, which confirms the formation of BCA-intercalated LDH.

**Figure 4:**
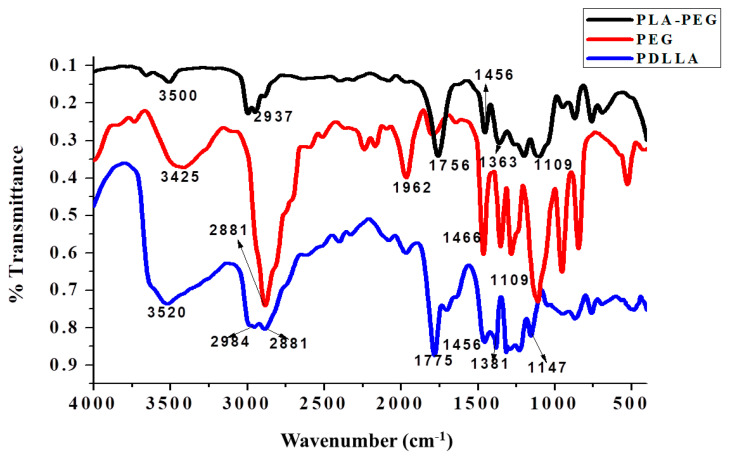
Overlay of the FTIR spectra of PLA-PEG (synthesized by transesterification), PDLLA, and PEG.

**Figure 5:**
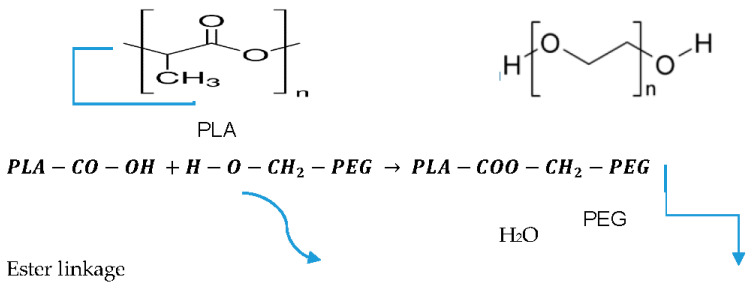
Transesterification reaction of PLA and PEG.

**Figure 6:**
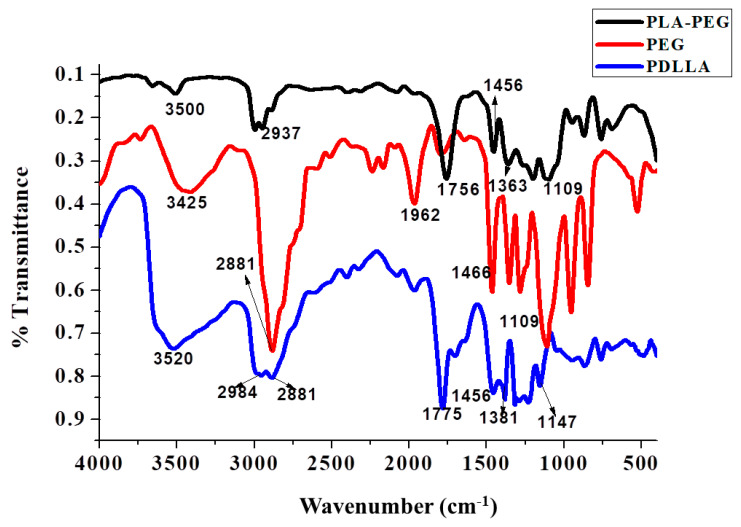
Overlay of the FTIR spectra of PLA-PEG copolymer (synthesized by acylation and esterification), PDLLA, and PEG.

The FTIR spectra of PDLLA, PEG, and PDLLA-PEG are represented in Figure 4 and Figure 5 (trans-esterification) and Figure 6 (acylation and esterification). In the FTIR of the copolymer, the peak at 3500 cm^−1^ is attributed to terminal OH^−1^ group, its intensity was reduced in PLA-PEG compared to homopolymer PEG, indicating the formation of an ester bond between terminal OH^−1^ groups. The intensification of ester peaks at 1756 cm^−1^ and 1456, 1109 cm^−1^ confirms the transesterification reaction, and the PLA-PEG copolymer was successfully synthesized [56].

**Figure 7:**
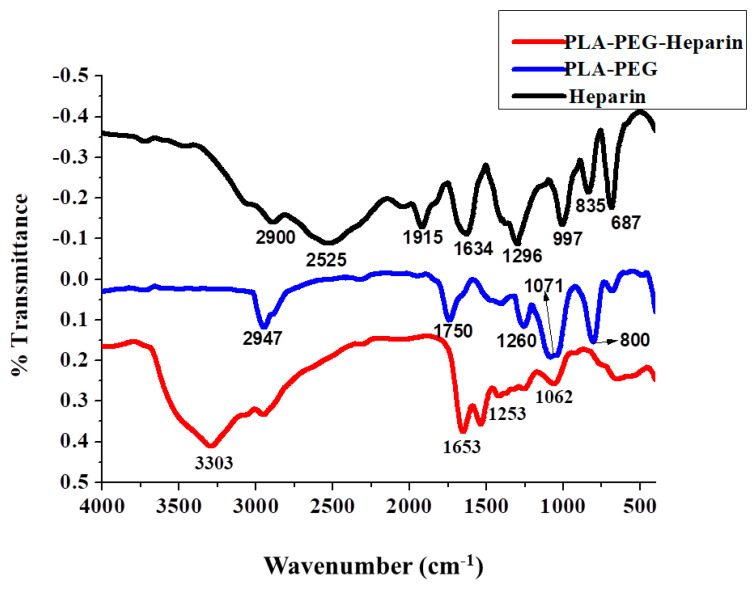
Overlay of the FTIR spectra of PLA-PEG, heparin, and PLA-PEG-heparin.

The FTIR spectra of PLA-PEG, heparin, and PLA-PEG-heparin are sho3wn in Figure 7. PLA-PEG-heparin was prepared by direct coupling reaction using DCC/DMAP, which utilizes the terminal hydroxyl groups of PLA-PEG and the carboxylic groups of heparin molecules. FTIR was used to confirm the heparin tagging. The broader absorption in the 3300-3500 cm^−1^ range is attributed to the characteristic N-H bond stretching absorption and/or hydrogen-bonded hydroxyl groups of PLA-PEG-heparin. In addition, IR spectra of PLA-PEG-heparin display the new absorption bands near 1250 and 1650 cm^−1^ as compared to that of PLA, which are characteristic absorption peaks of the sulfonated group (-SO3 stretching) and amino group (N-H bending) of conjugated heparin. These results indicate the formation of PLA-PEG-heparin [57].

### 2.2. XRD of Mg/Al (LDH) Nanoparticles

**Figure 8:**
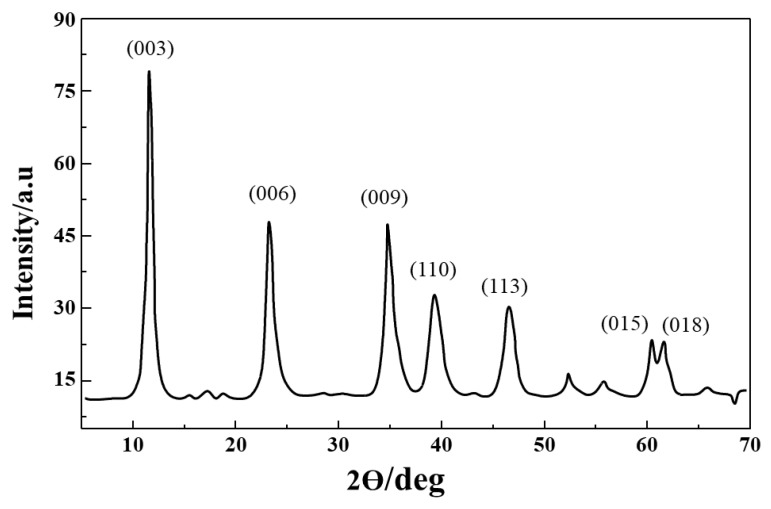
X-ray diffraction patterns of Mg/Al (LDH).

Figure 8 shows X-ray diffraction (XRD) patterns of dried Mg/Al LDH prepared by the co-precipitation method. Sharp and symmetric peaks appeared in the lower angle region, while broad and less symmetric peaks appeared in the region of higher angle due to (0 0 l) and (0 k l) reflections, respectively. From the peak indexing, it is clear that the ascribed structure is characterized as hexagonal unit cells with rhombohedral symmetry [58], and the whole pattern was matched with the JCPDS file. The d-spacing of the peak corresponding to (003) plane was around 0.76 nm, and the interlayer spacing was quite suitable for carbonate anions insertion. The diffraction peaks at $2\theta =12.3^{{}^{\circ}},\;23.8^{{}^{\circ}},\;35.2^{{}^{\circ}},\;39.6^{{}^{\circ}},\;46.8^{{}^{\circ}},\;52.6^{{}^{\circ}},60.5^{{}^{\circ}},61.8^{{}^{\circ}}$ correspond to the (003), (006), (009), (003), (110), (113), (015), and (018) planes, which confirmed the formation of LDH.

The average hydrodynamic diameter of the nanoparticles obtained from PCS (Figure S1 of Supporting Information) was found to be 241.1 nm, and the PDI was found to be 0.272, which is <1. Thus, it can be confirmed that the nanoparticles are less polydispersed. The zeta potential of Mg/Al (LDH) was shown in Figure S2 of the Supporting Information. It was found to be 26.7 mV at pH ≈7. At pH lower than the isoelectric point (pH of LDH ≈11.3), LDH nanoparticles were covered with a positively charged stern layer followed by a diffused electrical double layer. The brucite layers in Mg–Al LDH are itself positively charged. At pH 7, the substantially high value of zeta potential 26.7 mV indicates a significant amount of electrical double layer repulsion among the positively charged layers of LDH nuclei.

### 2.3. TEM Images of LDH

**Figure 9:**
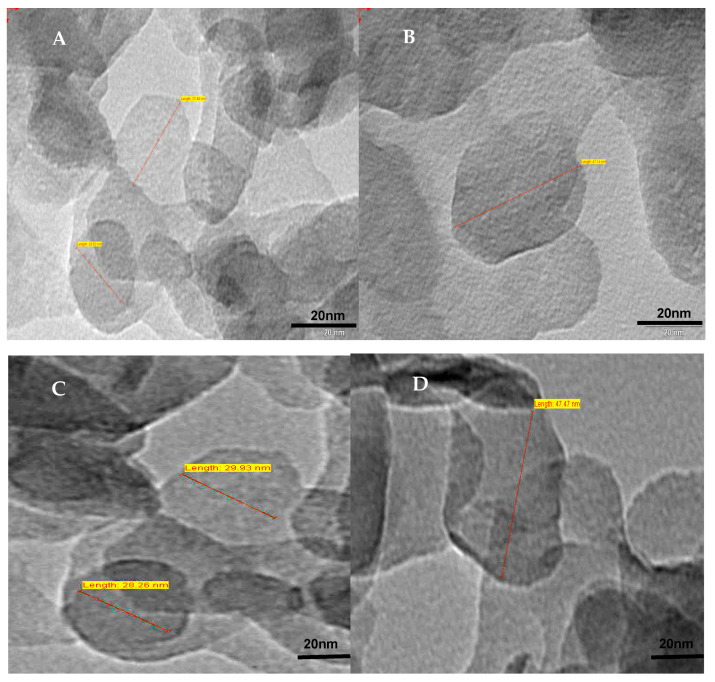
TEM images of Mg/Al (LDH) with hexagonal platelet structures of sizes: (**A**) 25 nm; (**B**) 50 nm; (**C**) 30 nm and (**D**) 45 nm.

Figure 9 represents the TEM images of LDH, which show a hexagonal platelet structure with uniform particle size of around 45 nm. The XRD and FTIR studies confirm the formation of LDH, and the TEM micrograph quantitatively shows its dimension.

**Figure 10:**
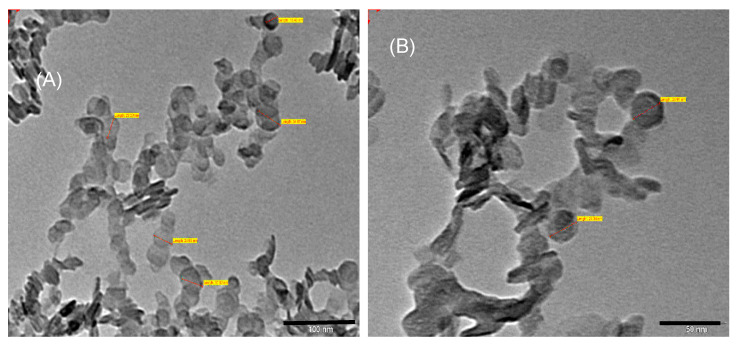
TEM images of (**A**) BCA-LDH-I and (**B**) BCA-LDH-C.

TEM images of BCA-LDH-I and BCA-LDH-C are depicted in Figure 10. There is no considerable difference in the size and platelet structure of particles after intercalation with BCA drug except that there is a slight increase in density after the incorporation of BCA into the LDH interlayers, which can be observed from the slight dark shade of the images compared to pristine LDH.

### 2.4. DSC Thermograms

**Figure 11:**
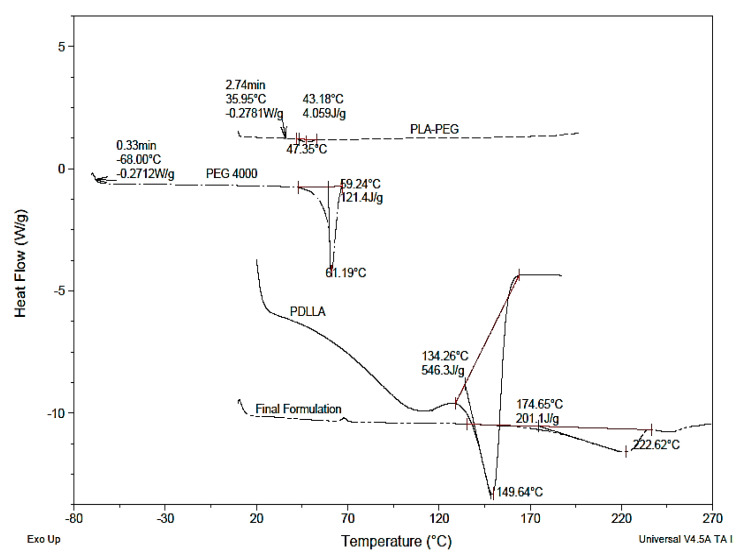
Overlay of DSC thermograms of PDLLA, PEG, PLA-PEG, and final formulation (PLA-PEG-heparin copolymer-encapsulated BCA-loaded LDH).

An overlay of DSC thermograms of PDLLA, PEG, PLA-PEG, and the final formulation (heparin-tagged PLA-PEG polymer-encapsulated BCA-loaded LDH) is represented in Figure 11. The DSC study revealed that the homopolymer samples of PDLLA and PEG produced sharp melting point (T_m_) peaks at 149.64 °C and 61.19 °C, respectively. The glass transition temperature (T_g_) of PDLLA and PEG was observed at 42 °C and −68 °C, respectively. The DSC study of PLA-PEG copolymer revealed a sharp melting point peak at 47.35 °C and glass transition temperature at 35.95 °C. No peaks pertaining to free PDLLA or PEG were noticed in the copolymer. This indicates that the copolymer is devoid of any homopolymer residues. A decrease in T_m_ as well as T_g_ has been observed on the copolymerization of PDLLA with PEG. As PEG is a plasticizer, it gets in between the polymer chains and spaces them apart from each other, increasing the free volume. This results in polymer chains sliding past each other more easily. As a result, the polymer chains can move around at lower temperatures, resulting in a decrease in the T_g_ of a polymer. In general, it is well documented that changes in the mechanical properties of solids (such as hardness, volume, percent elongation-to-break, and Young’s modulus) are affected by change in T_g_. In the case of stent coating, ideally, the polymer should have a T_g_ closer to body temperature. So, the polymer exists in the rubbery state and possesses ample flexibility and softness at body temperature. Such a polymer property for the stent coating prevents cracking and delamination after stent inflation [59].

In the final formulation, a shift in T_m_ to higher temperature (222.62 °C) as compared to PLA-PEG (149.64 °C) was observed. It can be attributed to the high T_m_ of BCA and LDH encapsulated in polymer. BCA and LDH were reported to have a sharp endothermic peak attributed to T_m_ at 213 °C and 430 °C, respectively [55]. T_g_ remained the same in both copolymer and final formulation.

### 2.5. Drug-Loading Efficiency

**Figure 12:**
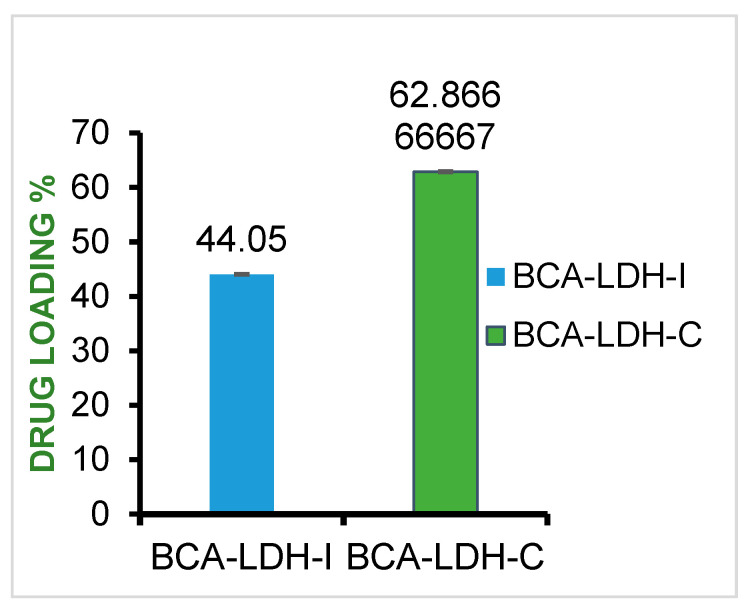
BCA loading percentage in BCA-LDH-I and BCA-LDH-C.

**Figure 13:**
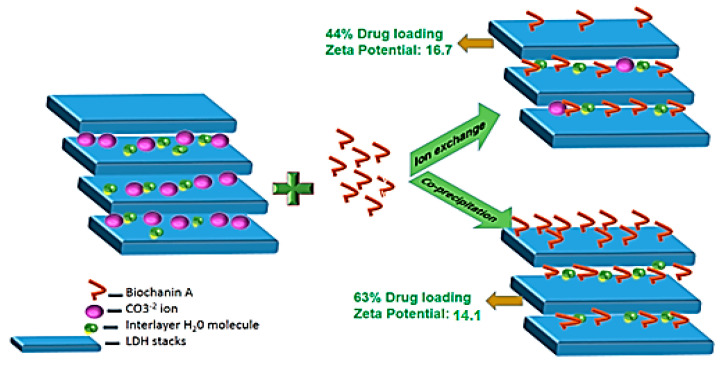
Schematic representation of drug loading in LDH nanoparticles by ion exchange and co-precipitation.

BCA was loaded in LDH nanoparticles by the ion exchange method and co-precipitation method with a mean drug loading of 44.05 and 63.8, respectively (Figure 12). The schematic of mechanism of drug loading is represented in Figure 13.

In the ion exchange method, a molecule (BCA) that is more electronegative than intercalated carbonate ion exchanges with it. Thus, comparatively less drug loading was observed through the ion exchange method, because in order to maintain the crystal structure of LDH, the carbonate ions were not all exchanged with drug.

In the co-precipitation method, during the synthesis of LDH, BCA was taken as the anion to be intercalated instead of the carbonate ion. So, drug loading was significantly greater than that of the ion exchange method. The zeta potential of BCA-LDH-C (14.7 mV) was less than that of BCA-LDH-I (17.1 mV); this might be due to the surface adhered drug by weak electrostatic interactions [55].

### 2.6. In Vitro Drug Release Study

**Figure 14:**
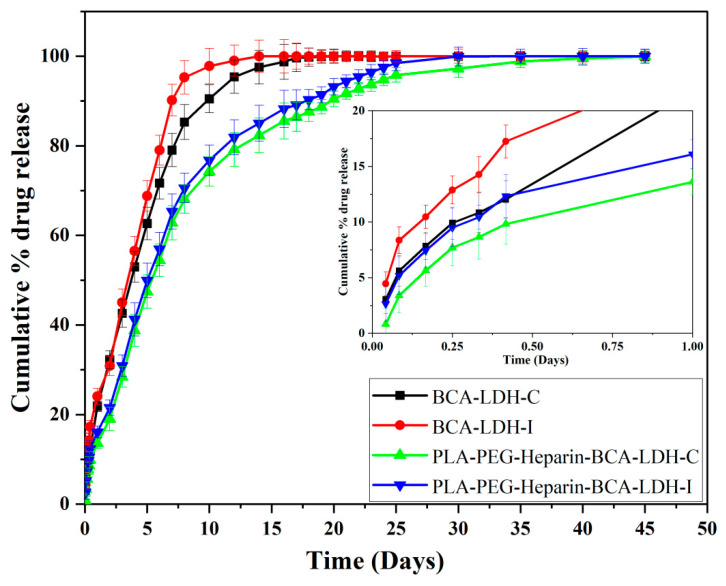
Cumulative drug release profile of BCA-LDH-C, BCA-LDH-I, PLA-PEG-heparin-BCA-LDH-C, and PLA-PEG-heparin-BCA-LDH-I in PBS (pH ≈7.4) for 50 days (n = 3), (inset displaying the release profile in 1st day).

The cumulative drug release profiles of BCA-LDH-C, BCA-LDH-I, PLA-PEG-heparin-BCA-LDH-I, and PLA-PEG-heparin-BCA-LDH-C in PBS at pH 7.4 are represented in Figure 14.

The release behavior of BCA drug from BCA-LDH-I nanoparticles, in PBS at pH 7.4, is presented in Figure 14. The release rate reached 24% after 24 h. Thereafter, a gradual increment of BCA was observed with release percentages of 45, 68, 90, and 99.9 after 3, 5, 7, and 14 days, respectively. The total drug was released within 14 days due to the weak electrostatic interaction between BCA molecules and the LDH.

The release of BCA from BCA-LDH-C nanoparticles is less than that of BCA-LDH-I at all the time points. In the first 24 h, only 21% of the drug was released, and then, 42, 62, 80, 95, and 99.9% of the drug release was observed at 3, 5, 7, 12, and 20 days, respectively. The release profile was almost linear until 10 days, and from 10 to 20 days, it was much slower and sustained. Therefore, it can be concluded that BCA-LDH-C shows a stability and consistency in its release profile. In order to maintain its crystal structure, ion exchange in BCA-LDH-C was bit slower, and the release was sustained for a much longer time compared to BCA-LDH-I.

The polymer-encapsulated nanoparticles are expected to release the drug at a much slower pace as compared to the pristine BCA-LDH nanoparticles. The overall 50 days BCA release profile of PLA-PEG-Heparin-BCA-LDH-C and PLA-PEG-Heparin-BCA-LDH-C nanohybrids at pH 7.4 showed very little burst release of <15% during the first 24 h, which can be attributed to the slower hydration of the polymer and release of the BCA that was adsorbed onto the surface of LDH particles. Subsequently, the polymer gets swollen and eroded, which exposes the LDH nanoparticles to the buffer and hastens the drug release, which may be attributed to the release of intercalated BCA in LDH and due to the anion exchange process between the intercalated anions in the interlayer and phosphate anions in the buffer.

The release of BCA from PLA-PEG-heparin-BCA-LDH-C was persistent and gradual, with release percentages of 13, 28, 47, 62, 74, 82, 90, and 99.9% after 1, 3, 5, 7, 10, 14, 20, and 45 days, respectively. The release process was close to linear up to 10 days, exponential from 10 to 20 days, and from 20 to 45 days, it was non-linear as a function of time. This could be attributed to the faster degradation rate of PLA-PEG-heparin copolymer during the first 2 weeks of study. In addition, the capability of the LDH framework to slow and control the release of the drug payload is appreciable.

Similarly, the release profile of PLA-PEG-heparin-BCA-LDH-I was faster than PLA-PEG-heparin-BCA-LDH-C, due to the weaker intercalation of BCA and lesser payload. The release trend was found to be 16, 30, 50, 65, 76, 85, 93, and 99.9% after 1, 3, 5, 7, 10, 12, 14, 20, and 30 days, respectively. The total drug was released within 30 days, whereas in PLA-PEG-heparin-BCA-LDH-C, the total drug was released in 45 days. Hence, PLA-PEG-heparin-BCA-LDH-C was showing a much more impressive and desirable drug release profile for the stent coating.

### 2.7. Mathematical Modelling

In order to further investigate the drug release mechanism, four pharmacokinetic models (zero-order, first-order, Higuchi, and Korsmeyer–Peppas) were fit to the experimentally obtained drug release profiles, the equations for which are mentioned below. Rigter and Peppas introduced the following empirical power equation based on which various mathematical models of drug release are derived [60].

$$Q=\frac{M_{t}}{M_{\infty }}\;;\;$$

$$\frac{M_{t}}{M_{\infty }}=kt^{n}\;$$

where *M_t_* and *M_∞_* are the absolute cumulative amounts of drug released at time *t* and at infinite time, respectively; *k* is a constant relating to the properties of the matrix and the drug, structural, and geometric characteristics of the device; and *n* is a dimensionless number. The exponent *n* describes the release kinetics, with *n* = 1.0 for zero-order kinetics.

Zero-order model:

*Q* = *kt*

First-order model:

*Q* = 1 − exp(−*kt*)

Higuchi model:

*Q* = *kt*^1/2^

Korsmeyer–Peppas model:

*Q* = *kt^n^*

where *k*, *Q*, *n*, and *t* are the kinetics constant, cumulative release ratio, diffusion exponent, and release time, respectively. The Korsmeyer–Peppas model was plotted for the initial 60% of release (*M_t_*/*M_∞_* ≤ 0.6), in which *M_t_* is the amount of drug released at time *t*, and *M_∞_* is the total amount of drug loaded. The *n* and *k* values were calculated from the slope and intercept of the plot of ln(*M_t_*/*M_∞_* ≤ 0.6) against ln *t*, respectively. Accordingly, the diffusion exponent (*n*) is used to indicate various release mechanisms. If the diffusion exponent (*n*) value is less than 0.5, release corresponds to quasi Fickian diffusion; if *n* = 0.5, the drug release corresponds to Case I or the Fickian diffusion mechanism in non-swellable matrices. If *n* = 0.5 to 1.0, the drug release corresponds to anomalous or non-Fickian transport, *n* = 1.0, the drug release corresponds to zero-order release or Case II transport, and if *n* > 1.0, the drug release corresponds to super Case II transport [60,61].

The mathematical modeling results of drug release kinetics are tabulated in Table 1 and Table 2. The release is most likely to be zero order with a regression coefficient of 0.996 for BC-LDH-C and 0.952 for BC-LDH-I. The PLA-PEG-heparin-BCA-LDH-C and PLA-PEG-heparin-BCA-LDH-I follows zero order with an R^2^ value of 0.912 and 0.906, respectively. However, the best fit model was Higuchi for both the polymer-encapsulated nanoparticles, which correspond to a slow and sustained release.

In order to further emphasize the release mechanism, the first 60% of the drug release was linearly plotted, and the “*n*” value obtained from the slope of the curve was found to be 0.889 and 0.901 for PLA-PEG-heparin-BCA-LDH-C and PLA-PEG-heparin-BCA-LDH-I, respectively. The release mechanism was anamolous non-Fickian transport accompanied by both diffusion and erosion of the matrix.

### 2.8. In Vitro Degradation Study of Polymer

**Figure 15:**
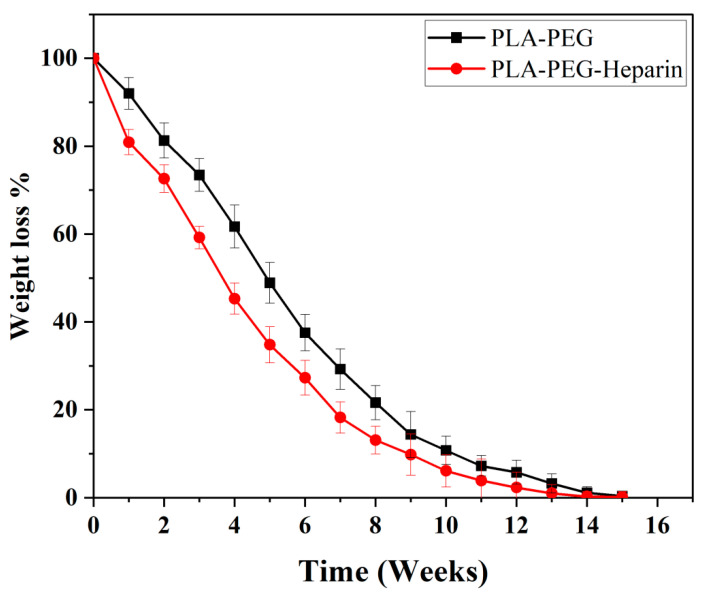
In vitro degradation profile of PLA-PEG and PLA-PEG-heparin copolymer.

The in vitro degradation profiles of the PLA-PEG and PLA-PEG-heparin copolymers are represented in Figure 15. The degradation of PLA-PEG-heparin was faster as compared to that of the PLA-PEG copolymer. As the degradation is by diffusion-controlled hydrolysis, initially, it takes some time for the diffusion of water molecules to penetrate into the polymeric matrix. PLA-PEG showed a stable and uniform degradation in the first week with <8% weight loss; then, 19, 40, 63, 78, 90, 95, and 99.7% weight loss was observed in 2nd, 4th, 6th, 8th, 10th, 12th, and 15th weeks, respectively. PLA-PEG-Heparin has displayed faster degradation with a weight loss of 20% in the first week followed by a weight loss of 28, 55, 70, 87, 94, 98, and 99.8% observed in the 2nd, 4th, 6th, 8th, 10th, 12th, and 15th weeks, respectively. It is very important for the drug coated on a stent to be completely eluted from polymeric matrix by 2–3 months post implantation, so as to avoid any inflammatory responses. The augmented initial degradation rate with PLA-PEG-heparin was due to the release of heparin moieties from the copolymer, which makes it more susceptible to hydrolytic cleavage. Hence, the degradation profile observed with PLA-PEG-heparin was found to be desirable profile for a DES coating.

### 2.9. Wettability Studies

**Figure 16:**
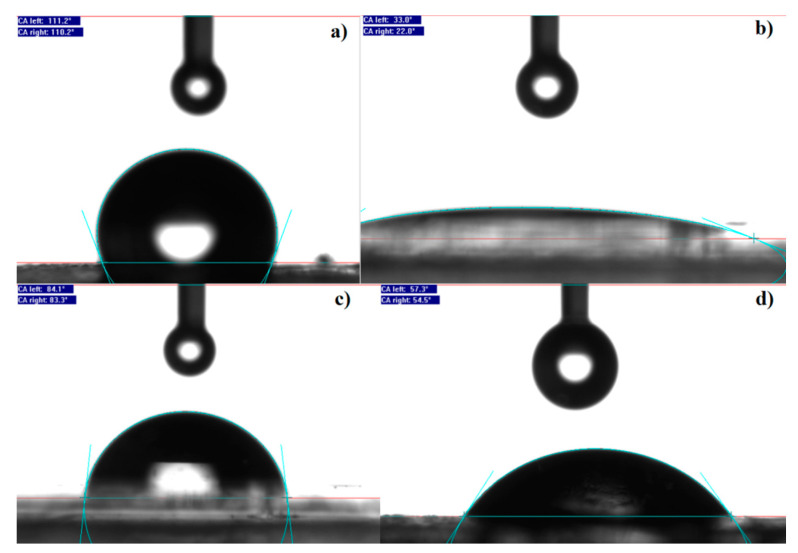
Wettability study of polymers (**a**) PLA, (**b**) PEG, (**c**) PLA-PEG, and (**d**) PLA-PEG-heparin.

The wetting properties of polymeric films were studied by measuring the water contact angle (CA). The contact angle results of polymers are represented in Figure 16. As such, PDLLA is hydrophobic with a contact angle of 110°, PEG is hydrophilic with a CA of 25°, whereas, PLA-PEG copolymer is intermediate between PDLLA and PEG with a CA of 84°. The CA of PLA-PEG-heparin was found to be 56°. So, it is optimum for a stent coating. It is desirable for a stent surface to be sufficiently hydrophilic to prevent the inflammatory responses. Aliphatic side chains of the amino acids of proteins orient toward hydrophobic surfaces, and more tend to adsorb. If the surface is hydrophobic, it is more prone to the adhesion of proteins. The interfacial layer wettability plays a significant role in the late stent thrombosis. Hence, the PLA-PEG-heparin copolymer was found to have desirable wetting properties that do not cause stent thrombosis [62,63].

### 2.10. Protein Adhesion Test

**Figure 17:**
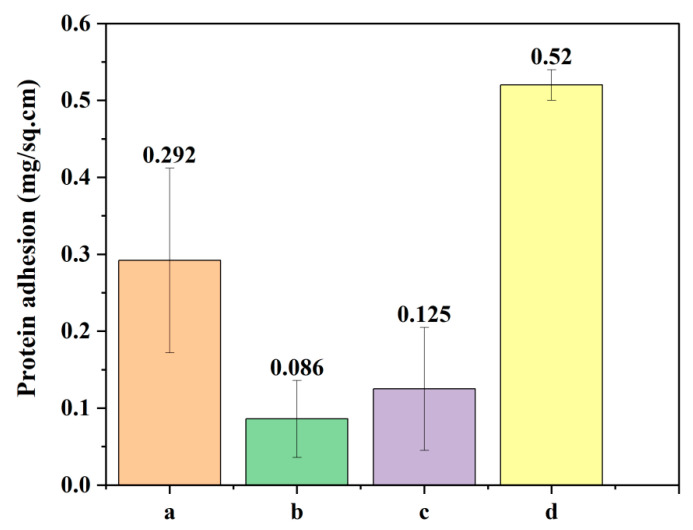
Protein adhesion test: (a) PLA, (b) PEG, (c) PLA-PEG, and (d) PLA-PEG-heparin.

Protein adhesion per square cm of PLA, PEG, PLA-PEG and PLA-PEG-heparin films were found to be 0.292, 0.086, 0.52, and 0.125, respectively (Figure 17). It is desirable that the polymeric surface should resist the adhesion of proteins to prevent thrombosis [64]. From the protein adhesion results, PLA-PEG and PLA-PEG-heparin copolymers were found to be less adherent surfaces to proteins.

### 2.11. In Vitro Hemocompatibility Study

**Figure 18:**
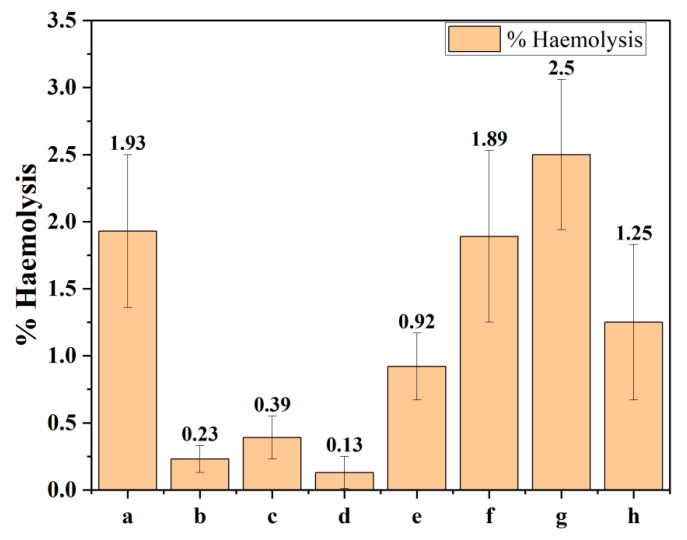
Hemolysis assay: (a) PLA, (b) PEG, (c) PLA-PEG, (d) PLA-PEG-heparin, (e) LDH (100 mg/mL), (f) LDH (200 mg/mL), (g) LDH (500 mg/mL), and (h) BCA-LDH-C (500 mg/mL).

The hemocompatibility results of PLA, PEG, PLA-PEG, LDH, and BCA-LDH-C are represented in Figure 18. The percentage of hemolysis of PLA, PEG, PLA-PEG, PLA-PEG-heparin, BCA-LDH-C (100 mg/mL), BCA-LDH-C (200 mg/mL), and BCA-LDH-C (500 mg/mL) was found to be 1.93, 0.13, 0.39, 0.13, 0.92, 1.89, and 2.5, respectively. The percentages of hemolysis of the polymers and LDH samples were found to be <5. Hence, the developed drug-eluting stent coating was found to be non-thrombogenic in nature.

**Figure 19:**
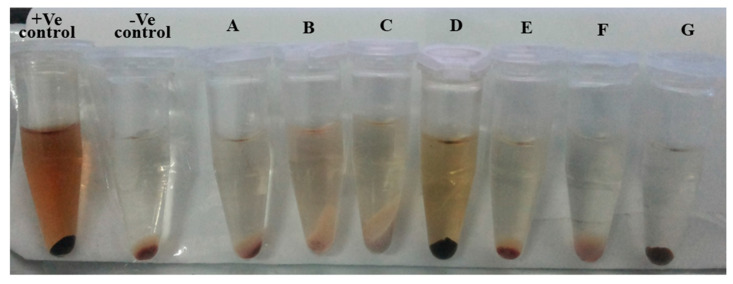
Optical images of hemolysis assay.

The optical images of the supernatant of each sample during hemolysis assay are represented in Figure 19. The intensity of color is the measure of degree of hemolysis that occurred in the presence of the sample. The positive control has the highest percentage of hemolysis (supernatant appears darker), and the negative has the least (supernatant appears clear). Samples A, B, and C represent the degree of hemolysis of LDH (200 mg/mL), LDH (500 mg/mL), and BCA-LDH-C (500 mg/mL), respectively. D, E, F, and G represent the percentage hemolysis of PLA, PEG, PLA-PEG, and PLA-PEG-heparin, respectively. A negligible percentage of hemolysis was observed from the PLA-PEG and PLA-PEG-heparin when compared to PLA alone. These results are in line with the quantitative hemolysis assay.

### 2.12. Stability Studies of BCA-LDH Nanoparticles

In the stability studies performed for 7 weeks with aqueous suspension of BCA-LDH-C at 37 °C and at a relative humidity of 75%, no considerable difference in zeta potential (Table 3) and average hydrodynamic diameter (Table 4) was noticed. Thus, the BCA-LDH-C nanoparticles were found to be stable for up to 7 weeks.

## 3. Discussion

In the current study, the loading efficiency achieved with the developed LDH nanoparticles is 65%; this is significantly greater than the earlier findings. The average size and stability of the synthesized LDH nanoparticles were found to be superior as compared to the existing reports [52]. The drug release profile was close to zero order with much greater control over the release profile due to the presence of dual barriers (LDH and the polymer), and a sustained release for up to 40 days was achieved. Achieving a slow and sustained, close to zero-order release is ideal release kinetics for a DES. In this study, we achieved close to zero-order release with anomalous non-Fickian diffusion where both diffusion and polymer erosion/relaxation play a role in the release. Most of the papers reported offered a burst release in the initial few hours, up to 30–90% drug were released in 24 h, and later, a slow and sustained release was displayed for about 10–20 days [65,66,67]. The release mechanism in most of the reports was based on polymer erosion, which accounts for the abrupt release of drug [68,69,70,71]. The degradation profile of the PLA-PEG-heparin copolymer was sustained and not abrupt, and complete degradation was achieved within 14 weeks. In the earlier reports, the degradation was delayed, which would evoke inflammatory responses to a greater extent [72,73]. To achieve a subtle balance between therapeutic dose and toxicity, the drug release kinetics is so critical. The degradation products should be non-thrombogenic, which have been achieved with the current study.

## 4. Materials and Methods

### 4.1. Materials

Magnesium nitrate hexahydrate [Mg(NO_3_)_2_·6H_2_O], aluminum nitrate nona hydrate [Al(NO_3_)_3_·9H_2_O], biochanin A, poly-(DL)-lactide (PDLLA) (MW: 1800–24,000 Da), and heparin sodium salt were purchased from Sigma Aldrich. Sodium Carbonate LR, Acetone LR, Polyethylene Glycol (PEG 4000 LR), Sodium Chloride AR ACS, Potassium Chloride AR, *di*-Sodium hydrogen orthophosphate anhydrous AR, Potassium dihydrogen orthophosphate AR, Thionyl chloride AR, Pyridine AR, Dicyclohexyl Carbodiimide (DCC) AR, and Dimethyl aminopyridine (DMAP) AR were purchased from SDFCL. Sodium Hydroxide, Methanol, Choloroform, Dichloromethane, Dimethyl formamide, n-hexane, and Ethyl acetate were purchased from Fisher Scientific. Ethanol was purchased from Shree Chalthan Vibhag Khand, Surat. Dialysis membrane-150 (MW~150,000) was purchased from Himedia, Mumbai. Milli-Q water was used in all experiments.

### 4.2. Methodology

#### 4.2.1. Synthesis

##### Synthesis of Mg/Al Layered Double Hydroxide (LDH) Nanoparticles

The co-precipitation method was used for preparing carbonate-intercalated Mg/Al LDH. Salt solution with a molar ratio of 2:1 was prepared by adding Mg(NO_3_)_2_·6H_2_O (2.2 mmol) and Al(NO_3_)_3_·9H_2_O (1.1 mmol) in 75 mL of deionized water. Na_2_CO_3_ solution (250 mL, 2.3 mmol) was prepared in deionized water and kept in a five-neck flat-bottom flask. The flask was fitted with two burettes, one condenser, one gas inlet–outlet, and one pH meter probe. The solution was degassed by purging nitrogen. Whole solution was stirred vigorously, and the previously prepared salt solution with a molar ratio of 2:1 Mg(NO_3_)_2_ 6H_2_O (2.2 mmol) and Al(NO_3_)_3_·9H_2_O (1.1 mmol) in 75 mL of deionized water was added dropwise in Na_2_CO_3_ solution. One molar NaOH solution was added dropwise simultaneously to the above solution to maintain constant pH (≈11). The whole solution was stirred at 60–65 °C for 12 h and a white-colored precipitate was separated from the solution by centrifugation (5000 rpm, 10 min, 25 °C) initially and then centrifuged thrice at 12,000 rpm, 10 min, at 25 °C, washed several times with deionized water, and dried in oven at 40 °C for 36 h.

##### Synthesis of PLA-PEG Copolymer

For the synthesis of PLA-PEG copolymer, PDLLA homopolymer with an average molecular weight (18,000–24,000 Daltons (determined by GPC)) and PEG 4000 were taken in 1:1 ratio. The homopolymers were dried in a hot air oven at 30 °C for 4 days and then stored in a desiccator until use. To achieve a homogeneous mixture, PDLLA and PEG were dissolved in acetone (50 mg of each polymer in 1 mL of acetone). Transesterification was performed in ampoule bottles. The polymer solution was placed into the bottle, and nitrogen was flowed through the solution continuously. The solvent was evaporated at 100 °C in approximately 2 h. The medium temperature was raised to 190 °C, and transesterification was conducted at this constant temperature for approximately 6 h under nitrogen atmosphere. Then, it was characterized and used for further studies.

##### Synthesis of PLA-PEG Copolymer by Acylation and Esterification Reaction

As the yield was less with transesterification reaction, another synthetic approach has been employed, which involves acylation followed by esterification.

Acyl Halide-terminated PLA-COCl (PLA-COCl) was synthesized first. For which carboxylic acid-terminated PLA had been flame-dried prior to use. PLA prepolymer was dissolved in anhydrous methylene chloride, and then, thionyl chloride and 1 wt % of DMF were added to the solution at 0 °C in an ice bath under N_2_ purging. The molar ratio of the PLA-COOH prepolymer to thionyl chloride was 1:2. The mixture was reacted at 60 °C for 4 h on Radley’s reactor in a fume hood. Finally, the methylene chloride and unreacted thionyl chloride were removed under reduced pressure [54].

For the synthesis of PLA-PEG diblock copolymer, PLA-COCl prepolymer was dissolved in anhydrous methylene chloride, and 1 molar ratio of PEG was introduced into the solution. Three different molar ratios of the PLA prepolymer to PEG have been synthesized, i.e., 50:50, 70:30, and 85:25. Anhydrous pyridine was added dropwise, while the temperature was maintained at 0 °C under a N_2_ environment. The reaction was carried out at room temperature for 12 h. Then, ice-chilled water was added to the reaction mixture and stirred for ½ h. It was transferred to a separating funnel, and the organic phase copolymer was collected. The polymer in organic phase was precipitated with n-hexane and ethylacetate; solvent was distilled by using a rotavapor. Then, it was washed 3 times with hexane/methylene chloride and dried in a vacuum oven at room temperature for 24 h [54].

##### Synthesis of Heparin-Tagged PLA-PEG Copolymer

Heparin-conjugated PLA-PEG was prepared by a direct coupling reaction using DCC and DMAP. Heparin (1.8 g, 1 × 10^−4^ mol) and PLA-PEG (0.5 g, 0.5 × 10^−4^ mol) were first dissolved in the mixture of formamide (50 mL) and *N*,*N*-dimethylformamide (DMF, 50 mL), respectively. DCC (1 × 10^−4^ mol) and DMAP (1 × 10^−4^ mol) were added to heparin solution with stirring for 10 min. Then, the PLA-PEG solution was dropped into the reaction solution with stirring at 50 °C for 12 h under nitrogen atmosphere. After the coupling reaction, the reaction solution was precipitated in excess methanol, and then, the precipitate was washed in distilled water. The precipitate was dissolved in chloroform, and the solution was precipitated in excess methanol. After filtering, the precipitate was dried at 35 °C for 24 h in a vacuum oven to eliminate the residual solvent.

#### 4.2.2. Analytical Method Development and Drug Loading in LDH Nanoparticles

##### Absorption Maxima Determination and Calibration Curve of Biochanin A

The UV-Visible measurement was performed using UV-1800, Shimadzu, operating in the spectral range of 190–500 nm. Biochanin A was dissolved in ethanol (1 mg/mL) and diluted with water. Spectrum was taken against ethanol as blank. The absorption maxima (λmax) was determined to be 263 nm. For the calibration curve, stock solution of 40 µg/mL of biochanin A was prepared in ethanol, from which serial dilutions of 1, 2, 3, 4, and 5 µg/mL were prepared in water in triplicate. The UV absorption of all the dilutions was taken against ethanol water mixture as a blank at 263 nm. The calibration curve and UV-VIS spectrum showing λmax are given in the Supporting Information.

##### Biochanin A (BCA) Loading in LDH Nanoparticles

BCA loading in pre synthesized Mg/Al(LDH) nanoparticles was done by ion exchange method (BCA-LDH-I). In this method, first, 0.001 M, 12.5 mL of BCA solution was prepared. Then, 3.55 mg of BCA was dissolved in ethanol and made up to 12.5 mL with water. After, 25 mg of LDH was added to it and magnetically stirred for 24 h at RT. Then, it was centrifuged at 14,000 RPM for 1 h at 25 °C. The supernatant was collected, and the UV-VIS absorbance of the supernatant was checked against the ethanol water mixture as blank to determine the percentage of drug loaded. Loading was done in triplicates, and the mean percentage drug loaded and Standard Deviation (SD) was calculated.

BCA loading with simultaneous synthesis of LDH nanoparticles was done by Co-precipitation method (BCA-LDH-C). This method was performed in a similar way as that of LDH nanoparticles. However, instead of Na_2_CO_3_, BCA solution was taken in a five-neck flask, so that BCA anions co-precipitate and replace the nitrate ions. Finally BCA-intercalated Mg/Al(LDH) nanoparticles were obtained and can be used for further characterization and release studies.

##### Nano-Encapsulation of BCA-LDH-C and BCA-LDH-I with PLA-PEG-Heparin

The solvent evaporation technique was used to stir a solution of copolymer in chloroform with BCA-LDH-C and BCA-LDH-I at 50 °C. The ratio of polymer to BCA-LDH was 1:2. The polymer-encapsulated BCA-LDH-C and BCA-LDH-I are named as PLA-PEG-heparin-BCA-LDH-C and PLA-PEG-heparin-BCA-LDH-I, respectively.

##### Drug Release Studies

In vitro drug release studies were performed in triplicates for BCA-LDH-C, BCA-LDH-I, PLA-PEG-heparin-BCA-LDH-C, and PLA-PEG-heparin-BCA-LDH-I by taking 22.8 mg of each powder in dialysis bags, and each bag was suspended in 200 mL of phosphate-buffered saline (PBS) of pH 7.4 in a sealed flask and shaken at 50 rpm in an incubator and shaker at 37 °C. An aliquot of 3 mL was withdrawn at regular intervals and replaced with 3 mL of fresh PBS to maintain sink conditions. The aliquots were centrifuged twice to remove possible nanoparticles. The concentration of released BCA in the aliquot sample was determined by measuring absorbance at λ = 262 nm and calculating the amount from the equation of the calibration curve.

#### 4.2.3. Characterizations

##### Functional Group Analysis by FTIR

Fourier transform infrared (FTIR) spectra of Mg/Al (LDH), biochanin A, BCA-LDH-I, BCA-LDH-C, PDLLA, PEG, PDLLA-PEG, PDLLA-PEG-heparin copolymer were recorded using the KBr pellet method. The respective samples were mixed with KBr in a ratio of 1:100, and the samples were analyzed using FTIR in absorbance mode at room temperature with wavenumber from 400 to 4000 cm^−1^ using a spectrophotometer (DRL 8000, Shimadzu analytical India Pvt. Ltd., Mumbai, India).

##### Zeta Potential and Hydrodynamic Diameter by Photon Correlation Spectroscopy

The average hydrodynamic diameter and PDI, zeta potential measurement of Mg/Al(LDH), Biochanin A, BCA-LDH-I, and BCA-LDH-C were performed at 25 °C using photon scattering, also known as the photon correlation spectroscopy (PCS) method in a Zetasizer, (Malvern Zetasizer NANO ZS 90, Malvern products, New Delhi, India) with dilute aqueous suspensions. The results are given in the supporting information.

##### Crystallite Structure by X-ray Diffraction

The structure and degree of carbonate intercalation of Mg/Al (LDH) were examined by an advance wide-angle X-ray diffractometer with CuKa radiation and a graphite monochromator (wavelength, k ¼ 0.154 nm, Bruker AXS D8, Germany, SICART, Gujarat, India). A thin sheet of the samples was placed on a quartz sample holder at room temperature and scanned at diffraction angle 2ϴ from 1° to 5° at a scan rate of 1°/min.

##### Particle Size and Structure by TEM 

The particle size and structure of carbonate anion-intercalated Mg/Al-LDH and BCA-LDH were determined through TEM analysis. The dispersion of 2 mg of each sample was prepared using a bath sonicator for 10 min and analysed by transmission electron microscopy (Tecnai 20, Phillips, Holland) by the copper grid method, which was performed at an operating voltage of 100 kV.

##### Thermal Analysis by Differential Scanning Calorimetry (DSC)

All measurements were carried out on an Indium-calibrated DSC Q20 V24.9 Build 121 (TA instruments, USA, Bangalore, India) provided with a refrigerated cooling system. Data were analyzed using universal analysis 2000 software (TA instruments, USA, Bangalore, India). Individual samples of PDLLA, PEG, PLA-PEG, and polymer-encapsulated BCA-loaded LDH (2–3 mg) were weighed directly on DSC aluminium pan and scanned between −60 and 300 °C at a heating rate of 10 °C/min in an atmosphere of dry nitrogen at 50 mL/min flow rate. Sample pans were crimped with lids. An empty pan with a lid kept in similar manner was used as reference. The DSC curves were overlaid for the evaluation of data.

#### 4.2.4. Wettability Study

The contact angles of PLA, PEG, PLA-PEG, and PLA-PEG-heparin films were measured by using a contact angle instrument (Data physics, OCA, Filderstadt, Germany) at a flow rate of 9.5 μL/s with a 0.52 mm needle by the sessile drop method. The average reading of each sample was taken and compared with the standard values [55].

#### 4.2.5. In Vitro Biodegradability Studies

Three samples each having 43 mg of PLA-PEG and PLA-PEG-heparin copolymer were placed in a test tube (predetermined weight) containing 5 mL PBS (pH 7.4) and placed in an orbital incubator and shaker at 37 °C, 50 rpm. An aliquot of the medium solution (5 mL) was withdrawn at certain time points and replaced with 5 mL of fresh PBS to maintain pH and sink conditions.The weight of the polymer in the test tube was noted at each time point by GPC and percentage of weight loss was calculated from the initial weight of polymer. The degradation profile of polymer was determined by plotting time vs. percentage weight loss curve; standard deviation was calculated [56]. The percentage of weight loss was calculated by using the following Equation (1):

(1) $$\mathrm{Weight}\;\mathrm{loss}\;\left(\%\right)=\frac{\mathrm{Initial}\;\mathrm{Weight}-\mathrm{final}\;\mathrm{weight}}{\mathrm{initial}\;\mathrm{weight}}\times 100.$$

#### 4.2.6. Hemocompatability Study

A hemocompatability study of LDH, BCA-LDH-C, PLA, PEG, PLA-PEG, and PLA-PEG-heparin was performed by the previously reported method [57,58]. Acid citrate dextrose (ACD) solution was used as an anticoagulant agent for blood where citrate acts as an anticoagulant and chelating agent for blood. Phosphate buffer saline was (PBS 7.4 pH) used to maintain the pH of solution similar to that of the natural pH of blood. For the preparation of ACD, citric acid (365 mg), anhydrous tri-sodium citrate (110 mg), and monohydrate dextrose (1225 mg) was mixed in water, and the volume was made up to 5 mL. For the preparation of PBS (7.4 pH), 800 mg of sodium chloride, 20 mg of potassium chloride, 144 mg of di sodium hydrogen phosphate, and 24 mg of potassium di hydrogen phosphate was mixed in a beaker, and the volume was made up to 100 mL. The pH of buffer was adjusted to pH 7.4.

Then, 100 mg/mL of each sample of PLA, PEG, PLA-PEG, PLA-PEG-heparin; LDH at concentrations of 100, 200 and 500 mg/mL; and BCA-LDH-C (500 mg/mL) were taken in triplicates, while 0.5 mL ACD solution was dissolved in 4.5 mL blood. The samples were soaked in a separate beaker in PBS buffer, and it was kept at 37 °C for 24 h. To make the positive control, 0.5 mL of ACD blood was dissolved in 3 mL of water, and for negative control, 0.5 mL of ACD blood was dissolved in 3 mL of PBS (7.4 pH). All the samples, positive control and negative control, were incubated at 37 °C for 2 h. Then, samples were removed, and each sample was centrifuged at 2000 rpm for 15 min. The supernatant of each sample was collected, and absorbance was measured at 540 nm by U.V. Spectrophotometer (Shimadzu UV-1800, Shimadzu Analytical (India) Pvt. Ltd., Mumbai, India). The percentage of hemolysis in both PCL and PLA were calculated by using the following Equation (2).

(2) $$\%\;\mathrm{of}\;\mathrm{haemolyis}=\frac{O.D\;\mathrm{of}\;\mathrm{sample}-O.D\;\mathrm{of}\;\mathrm{negative}\;\mathrm{control}\;}{O.D\;\mathrm{of}\;\mathrm{positive}\;\mathrm{control}-O.D\;\mathrm{of}\;\mathrm{negative}\;\mathrm{control}\;}\times 100$$

The nature of materials was decided by comparing with reported standards [58]. The material is considered as non-hemolytic if the percentage of hemolysis is <2%, hemolytic within an acceptable limit if the percentage of hemolysis is between 2 and 5%; whereas the material becomes hemolytic and unacceptable if the limit crosses above 5%.

#### 4.2.7. Protein Adhesion Test

It was performed by the Folin–Lowry method, which was reported elsewhere [59]. The standard plot of BSA was determined first. For tests samples, 1 cm^2^ films of PLA, PEG, PLA-PEG, and PLA-PEG-heparin were equilibrated in PBS for ½ h and then incubated in BSA solution for 1 h. UV absorbance of supernatant was recorded at 660 nm. The amount of protein adhered was calculated from a standard plot.

#### 4.2.8. Stability Studies of BCA-LDH Nanoparticles:

Aqueous suspensions of BCA-LDH nanoparticles were analyzed for change in size as well as zeta potential for 7 weeks by using Malvern Zeta sizer nano ZS.

### 4.3. Statistical Analysis

For statistical analysis, triplicate data are presented as mean ± SD and were analysed by ANOVA using Origin Pro 9.0 software, LaGa Systems Pvt Ltd., Hyderabad, India. *p* < 0.05 is considered statistically significant.

**Figure 20:**
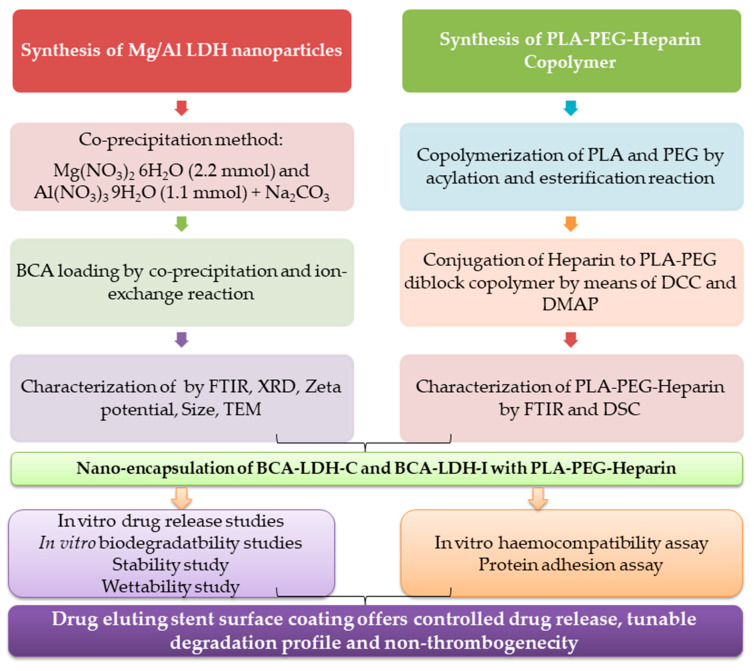
Schematic of the work flow.

A schematic of the work flow is illustrated in Figure 20 for easy following and understanding the work and the results.

## 5. Conclusions

Mg/Al (LDH) nanoparticles have been successfully synthesized and were confirmed by FTIR, XRD, TEM, and zeta potential. The nanoparticles synthesized were uniform with a particle size of 45 nm and a zeta potential of 27.2 mV. Significant drug loading (63%) has been observed in LDH nanoparticles with a desirable controlled drug-release profile up to 45 days. PLA-PEG copolymer and heparin-tagged PLA-PEG copolymer have been successfully synthesized and were confirmed by FTIR. The complete degradation of PLA-PEG-heparin copolymer has been observed within 14 weeks. Nano-encapsulation of BCA-LDH has been successfully done for the final stent coating. Zero-order release was observed for BCA-LDH-I and BCA-LDH-C samples. Encapsulation with heparin-tagged PLA-PEG copolymer resulted in a more sustained release. Further, mathematical modeling of release kinetics revealed the release mechanism to be anamolous non-Fickian diffusion with a close to zero-order profile. The developed stent surface coating is non-thrombogenic, anti-fouling, and sufficiently hydrophilic and releases the drug in a controlled manner with a desirable polymer degradation profile of 14 weeks. Therefore, it can be concluded that the developed stent surface coating offers controlled drug release, a tunable degradation profile, non-thrombogenecity, and may prevent restenosis without delaying re-endothelialization.

## Figures and Tables

**Table 1:** Regression coefficients (R^2^) obtained by fitting of drug release in kinetic models.

Models			R^2^ Values		
		BCA-LDH-I	BCA-LDH-C	PLA-PEG-Heparin-BCA-LDH-I	PLA-PEG-Heparin-BCA-LDH-C
Zero-order		0.952	0.996	0.906	0.912
		(First 60%)	(First 60%)	(First 60%)	(First 60%)
		0.901 (overall)	0.859 (overall)	0.845 (overall)	0.878 (overall)
First-order		0.730	0.793	0.815	0.762
Higuchi		0.825	0.898	0.932	0.955
Korsmeyer–Peppas (Mt/M∞ ≤ 0.6)	R^2^	0.912	0.917	0.889	0.901
	*n*	0.92	1.11	0.85	0.79

**Table 2:** Release coefficient of mathematical model fitting of drug release kinetics.

Models		Release Coefficient (*k*)		
	BCA-LDH-I	BCA-LDH-C	PLA-PEG-Heparin-BCA-LDH-I	PLA-PEG-Heparin-BCA-LDH-C
Zero-order	13.1	12.2	−4.3	−3.5
First-order	1.4	1.5	2.7	2.9
Higuchi	2.77	2.65	0.975	0.955
Korsmeyer–Peppas (*M_t_*/*M_∞_* ≤ 0.6)	0.35	0.41	1.23	1.12

**Table 3:** Zeta potential of BCA-LDH-C nanoparticles for 7 weeks.

Time (In Weeks)	Zeta Potential (mV)			Mean Zeta Potential (mV)
	i	ii	iii	
1	15.1	15.0	15.2	15.1
2	15	15.1	14.9	15
3	15	14.9	15.1	15
4	14.7	14.9	14.8	14.7
5	14.8	14.9	14.7	14.8
6	14.9	15	14.8	14.9
7	14.8	14.7	14.9	14.8

**Table 4:** Particle size of BCA-LDH-C for 7 weeks.

Time (In Weeks)	Size (nm)			Average (nm)	PDI
	i	ii	iii		
1	241.1	241.0	241.2	241.1	0.272
2	244.1	244.0	244.2	244.1	0.253
3	248.1	248.3	248.0	248.2	0.241
4	250.1	250.0	250.3	250.1	0.220
5	250.1	250.3	250.1	250.2	0.213
6	255.5	255.6	255.5	255.5	0.261
7	258.3	258.4	258.3	258.3	0.274

## Supplementary Materials

The following are available online at https://www.mdpi.com/article/10.3390/ijms22115433/s1, Figure S1. Hydrodynamic diameter and Polydispersity index (PDI) of aqueous suspension of LDH nanoparticles, Figure S2. Zeta potential of Mg/Al (LDH), Figure S3. Zeta potential of Biochanin A, Figure S4. Zeta potential of BCA-LDH-I, Figure S5. Zeta potential of BCA-LDH-C, Figure S6. UV-Visible spectrum of Biochanin A, Figure S7. Calibration curve of Biochanin A.

## Abbreviations

CAD	Coronary Artery Disease
CVD	Cardio Vascular Disease
PCI	Percutaneous Coronary Intervention
ISR	In Stent Restenosis
VSMC	Vascular Smooth Muscle Cells
EC	Endothelial Cells
NH	Neointimal Hyperplacia
PLA-PEG	Poly lactic acid–co-Poly ethylene glycol
BMS	Bare Metal Stents
DES	Drug-eluting stent
LDH	Layered Double Hydroxide
BCA	Biochanin A
PLA	Poly-DL-Lacticacid
PEG	Polyethylene glycol
PLA-PEG	Polylactide-co-polyethylene glycol
PLA-PEG-Heparin	Heparin-tagged Polylactide-co-polyethylene glycol
BCA-LDH	Biochanin A-loaded LDH
BCA-LDH-I	Biochanin A-loaded LDH nanoparticles produced by ion-exchange method
BCA-LDH-C	Biochanin A-loaded LDH nanoparticles produced by co-precipitation method
PLA-PEG-Heparin-BCA-LDH-I	Biochanin A-loaded LDH nanoparticles produced by ion-exchange method encapsulated in PLA-PEG-Heparin copolymer
PLA-PEG-Heparin-BCA-LDH-C	Biochanin A-loaded LDH nanoparticles produced by co-precipitation method encapsulated in PLA-PEG-Heparin copolymer
BSA	Bovine serum albumin
ACD	Acid Citrate Dextrose

## References

[1] Butt M., Connolly D., Lip G.Y. (2009). Drug-eluting stents: A comprehensive appraisal. Future Cardiol..

[2] Costa M.A., Simon D.I. (2005). Molecular Basis of Restenosis and Drug-Eluting Stents. Circulation.

[3] Martin D.M., Boyle F.J. (2011). Drug-eluting stents for coronary artery disease: A review. Med. Eng. Phys..

[4] Inoue T., Croce K., Morooka T., Sakuma M., Node K., Simon D.I. (2011). Vascular Inflammation and Repair: Implications for Re-Endothelialization, Restenosis, and Stent Thrombosis. JACC Cardiovasc. Interv..

[5] Simard T., Hibbert B., Ramirez F.D., Froeschl M., Chen Y.-X., O’Brien E.R. (2014). The Evolution of Coronary Stents: A Brief Review. Can. J. Cardiol..

[6] Hayashi S.-I., Yamamoto A., You F., Yamashita K., Ikegame Y., Tawada M., Yoshimori T., Shimizu S., Nakashima S. (2009). The Stent-Eluting Drugs Sirolimus and Paclitaxel Suppress Healing of the Endothelium by Induction of Autophagy. Am. J. Pathol..

[7] Park K. (2012). Dual drug-eluting stent. J. Control. Release.

[8] Finn A.V., Kolodgie F.D., Harnek J., Guerrero L., Acampado E., Tefera K., Skorija K., Weber D.K., Gold H.K., Virmani R. (2005). Differential Response of Delayed Healing and Persistent Inflammation at Sites of Overlapping Sirolimus- or Paclitaxel-Eluting Stents. Circulation.

[9] Zimarino M., Corazzini A., Ricci F., Di Nicola M., De Caterina R. (2013). Late thrombosis after double versus single drug-eluting stent in the treatment of coronary bifurcations: A meta-analysis of randomized and observational studies. JACC Cardiovasc. Interv..

[10] Ceylan H., Tekinay A.B., Guler M.O. (2011). Selective adhesion and growth of vascular endothelial cells on bioactive peptide nanofiber functionalized stainless steel surface. Biomaterials.

[11] Chen J.P., Hou D., Pendyala L., Goudevenos J.A., Kounis N.G. (2009). Drug-Eluting Stent Thrombosis: The Kounis Hypersensitivity-Associated Acute Coronary Syndrome Revisited. JACC Cardiovasc. Interv..

[12] Massberg S., Byrne R.A., Kastrati A., Schulz S., Pache J., Hausleiter J., Ibrahim T., Fusaro M., Ott I., Schömig A. (2011). Polymer-free sirolimus-and probucol-eluting versus new generation zotarolimus-eluting stents in coronary artery disease: The Intracoronary Stenting and Angiographic Results: Test Efficacy of Sirolimus-and Probucol-Eluting versus Zotarolimus-eluting Stents (ISAR-TEST 5) trial. Circulation.

[13] Kim W., Jeong M.H., Cha K.S., Hyun D.W., Hur S.H., Kim K.B., Hong Y.J., Park H.W., Kim J.H., Ahn Y.K. (2005). Effect of Anti-Oxidant (Carvedilol and Probucol) Loaded Stents in a Porcine Coronary Restenosis Model. Circ. J..

[14] Houston S.A., Ugusman A., Gnanadesikan S., Kennedy S. (2016). An investigation of the antiplatelet effects of succinobucol (AGI-1067). Platelets.

[15] Dourron H.M., Jacobson G.M., Park J.L., Liu J., Reddy D.J., Scheel M.L., Pagano P.J. (2005). Perivascular gene transfer of NADPH oxidase inhibitor suppresses angioplasty-induced neointimal proliferation of rat carotid artery. Am. J. Physiol. Circ. Physiol..

[16] Bräsen J.H., Leppänen O., Inkala M., Heikura T., Levin M., Ahrens F., Rutanen J., Pietsch H., Bergqvist D., Levonen A.-L. (2007). Extracellular Superoxide Dismutase Accelerates Endothelial Recovery and Inhibits In-Stent Restenosis in Stented Atherosclerotic Watanabe Heritable Hyperlipidemic Rabbit Aorta. J. Am. Coll. Cardiol..

[17] Nakazawa G., Granada J.F., Alviar C.L., Tellez A., Kaluza G.L., Guilhermier M.Y., Parker S., Rowland S.M., Kolodgie F.D., Leon M.B. (2010). Anti-CD34 Antibodies Immobilized on the Surface of Sirolimus-Eluting Stents Enhance Stent Endothelialization. JACC Cardiovasc. Interv..

[18] Kleinedler J.J., Foley J.D., Alexander J.S., Roerig S.C., Hebert V.Y., Dugas T.R. (2011). Synergistic effect of resveratrol and quercetin released from drug-eluting polymer coatings for endovascular devices. J. Biomed. Mater. Res. Part B Appl. Biomater..

[19] Kleinedler J., Foley J., Dugas T. (2009). Cytotoxicity and efficacy evaluation of polymeric nanoparticles containing resveratrol and quercetin for use on drug eluting stents. FASEB J..

[20] Yang J., Zeng Y., Zhang C., Chen Y.-X., Yang Z., Li Y., Leng X., Kong D., Wei X.-Q., Sun H.-F. (2013). The prevention of restenosis in vivo with a VEGF gene and paclitaxel co-eluting stent. Biomaterials.

[21] Naghavi N., De Mel A., Alavijeh O.S., Cousins B.G., Seifalian A.M. (2013). Nitric Oxide Donors for Cardiovascular Implant Applications. Small.

[22] Majewska P., Oledzka E., Sobczak M. (2020). Overview of the latest developments in the field of drug-eluting stent technology. Biomater. Sci..

[23] Ryu S.K., Mahmud E., Tsimikas S. (2009). Estrogen-Eluting Stents. J. Cardiovasc. Transl. Res..

[24] New G., Moses J.W., Roubin G.S., Leon M.B., Colombo A., Iyer S.S., Tio F.O., Mehran R., Kipshidze N. (2002). Estrogen-eluting, phosphorylcholine-coated stent implantation is associated with reduced neointimal formation but no delay in vascular repair in a porcine coronary model. Catheter. Cardiovasc. Interv..

[25] Airoldi F., Di Mario C., Ribichini F., Presbitero P., Sganzerla P., Ferrero V., Vassanelli C., Briguori C., Carlino M., Montorfano M. (2005). 17-Beta-Estradiol Eluting Stent Versus Phosphorylcholine-Coated Stent for the Treatment of Native Coronary Artery Disease. Am. J. Cardiol..

[26] Abizaid A., Albertal M., Costa M.A., Abizaid A.S., Staico R., Feres F., Mattos L.A., Sousa A.G., Moses J., Kipshidize N. (2004). First human experience with the 17-beta-estradiol–eluting stent: The estrogen and stents to eliminate restenosis (EASTER) trial. J. Am. Coll. Cardiol..

[27] Schrepfer S., Deuse T., Münzel T., Schäfer H., Braendle W., Reichenspurner H. (2006). The selective estrogen receptor-β agonist biochanin A shows vasculoprotective effects without uterotrophic activity. Menopause.

[28] Kumar T., Sharma M., Rana A., Lingaraju M.C., Parida S., Kumar D., Singh T.U. (2020). Biochanin-A elicits relaxation in coronary artery of goat through different mechanisms. Res. Veter. Sci..

[29] Somjen D., Knoll E., Kohen F., Stern N. (2001). Effects of phytoestrogens on DNA synthesis and creatine kinase activity in vascular cells. Am. J. Hypertens..

[30] Puranik A.S., Dawson E.R., Peppas N.A. (2013). Recent advances in drug eluting stents. Int. J. Pharm..

[31] Kolandaivelu K., Swaminathan R., Gibson W.J., Kolachalama V.B., Nguyen-Ehrenreich K.-L., Giddings V.L., Coleman L., Wong G.K., Edelman E.R. (2011). Stent thrombogenicity early in high-risk interventional settings is driven by stent design and deployment and protected by polymer-drug coatings. Circulation.

[32] Strohbach A., Busch R. (2015). Polymers for Cardiovascular Stent Coatings. Int. J. Polym. Sci..

[33] Yazdani S.K., Sheehy A., Pacetti S., Rittlemeyer B., Kolodgie F.D., Virmani R. (2016). Stent Coating Integrity of Durable and Biodegradable Coated Drug Eluting Stents. J. Interv. Cardiol..

[34] Rizas K.D., Mehilli J. (2016). Stent Polymers. Circ. Cardiovasc. Interv..

[35] Stefanini G.G., Byrne R.A., Serruys P.W., De Waha A., Meier B., Massberg S., Jüni P., Schömig A., Windecker S., Kastrati A. (2012). Biodegradable polymer drug-eluting stents reduce the risk of stent thrombosis at 4 years in patients undergoing percutaneous coronary intervention: A pooled analysis of individual patient data from the ISAR-TEST 3, ISAR-TEST 4, and LEADERS randomized trials. Eur. Heart J..

[36] Tada N., Virmani R., Grant G., Bartlett L., Black A., Clavijo C., Christians U., Betts R., Savage D., Su S.-H. (2010). Polymer-Free Biolimus A9-Coated Stent Demonstrates More Sustained Intimal Inhibition, Improved Healing, and Reduced Inflammation Compared With a Polymer-Coated Sirolimus-Eluting Cypher Stent in a Porcine Model. Circ. Cardiovasc. Interv..

[37] Ormiston J.A., Serruys P.W., Regar E., Dudek D., Thuesen L., Webster M.W., Onuma Y., Garcia-Garcia H.M., McGreevy R., Veldhof S. (2008). A bioabsorbable everolimus-eluting coronary stent system for patients with single de-novo coronary artery lesions (ABSORB): A prospective open-label trial. Lancet.

[38] Bae I.-H., Park I.-K., Park D.S., Lee H., Jeong M.H. (2012). Thromboresistant and endothelialization effects of dopamine-mediated heparin coating on a stent material surface. J. Mater. Sci. Mater. Electron..

[39] Yang Z., Yang Y., Zhang L., Xiong K., Li X., Zhang F., Wang J., Zhao X., Huang N. (2018). Mussel-inspired catalytic selenocystamine-dopamine coatings for long-term generation of therapeutic gas on cardiovascular stents. Biomaterials.

[40] Luo R., Tang L., Zhong S., Yang Z., Wang J., Weng Y., Tu Q., Jiang C., Huang N. (2013). In Vitro Investigation of Enhanced Hemocompatibility and Endothelial Cell Proliferation Associated with Quinone-Rich Polydopamine Coating. ACS Appl. Mater. Interfaces.

[41] Hou R., Wu L., Wang J., Yang Z., Tu Q., Zhang X., Huang N. (2019). Surface-Degradable Drug-Eluting Stent with Anticoagulation, Antiproliferation, and Endothelialization Functions. Biomolecules.

[42] Lockwood N.A., Hergenrother R.W., Patrick L.M., Stucke S.M., Steendam R., Pacheco E., Virmani R., Kolodgie F.D., Hubbard B. (2010). In Vitro and In Vivo Characterization of Novel Biodegradable Polymers for Application as Drug-Eluting Stent Coatings. J. Biomater. Sci. Polym. Ed..

[43] Steele T.W., Huang C.L., Widjaja E., Boey F.Y., Loo J.S., Venkatraman S.S. (2011). The effect of polyethylene glycol structure on paclitaxel drug release and mechanical properties of PLGA thin films. Acta Biomater..

[44] Garg A., Sharma R., Pandey V., Patel V., Yadav A.K. (2017). Heparin-Tailored Biopolymeric Nanocarriers in Site-Specific Delivery: A Systematic Review. Crit. Rev. Ther. Drug Carr. Syst..

[45] Chawla A.S., Chang T.M.S. (1985). In-Vivo Degradation of Poly(Lactic Acid) of Different Molecular Weights. Biomater. Med. Devices Artif. Organs.

[46] Sakiyama-Elbert S.E. (2014). Incorporation of heparin into biomaterials. Acta Biomater..

[47] Pugazhendhi A., Edison T.N.J.I., Karuppusamy I., Kathirvel B. (2018). Inorganic nanoparticles: A potential cancer therapy for human welfare. Int. J. Pharm..

[48] Baeza A., Ruiz-Molina D., Vallet-Regí M. (2017). Recent advances in porous nanoparticles for drug delivery in antitumoral applications: Inorganic nanoparticles and nanoscale metal-organic frameworks. Expert Opin. Drug Deliv..

[49] Chatterjee A., Bharadiya P., Hansora D. (2019). Layered double hydroxide based bionanocomposites. Appl. Clay Sci..

[50] Ladewig K., Xu Z.P., Lu G.Q. (2009). (Max) Layered double hydroxide nanoparticles in gene and drug delivery. Expert Opin. Drug Deliv..

[51] Li L., Gu W., Chen J., Chen W., Xu Z.P. (2014). Co-delivery of siRNAs and anti-cancer drugs using layered double hydroxide nanoparticles. Biomaterials.

[52] Kuthati Y., Kankala R.K., Lee C.-H. (2015). Layered double hydroxide nanoparticles for biomedical applications: Current status and recent prospects. Appl. Clay Sci..

[53] Bi X., Zhang H., Dou L. (2014). Layered Double Hydroxide-Based Nanocarriers for Drug Delivery. Pharmaceutics.

[54] Sohrabnezhad S., Poursafar Z., Asadollahi A. (2020). Synthesis of novel core@shell of MgAl layered double hydroxide @ porous magnetic shell (MgAl-LDH@PMN) as carrier for ciprofloxacin drug. Appl. Clay Sci..

[55] Chakraborty J., Roychowdhury S., Sengupta S., Ghosh S. (2013). Mg–Al layered double hydroxide–methotrexate nanohybrid drug delivery system: Evaluation of efficacy. Mater. Sci. Eng. C.

[56] Zhao H., Liu Z., Park S.-H., Kim S.-H., Kim J.-H., Piao L. (2012). Preparation and Characterization of PEG/PLA Multiblock and Triblock Copolymer. Bull. Korean Chem. Soc..

[57] Jee K.S., Park H.D., Park K.D., Kim Y.H., Shin J.-W. (2004). Heparin Conjugated Polylactide as a Blood Compatible Material. Biomacromolecules.

[58] Anjum M.J., Zhao J., Asl V.Z., Yasin G., Wang W., Wei S., Zhao Z., Khan W.Q. (2019). In-situ intercalation of 8-hydroxyquinoline in Mg-Al LDH coating to improve the corrosion resistance of AZ31. Corros. Sci..

[59] Jadhav N.R., Gaikwad V.L., Nair K.J., Kadam H.M. (2009). Glass transition temperature: Basics and application in pharmaceutical sector. Asian J. Pharm..

[60] Ritger P.L., Peppas N.A. (1987). A simple equation for description of solute release II. Fickian and anomalous release from swellable devices. J. Control. Release.

[61] Paarakh M.P., Jose P.A., Setty C., Christoper G. (2018). Release kinetics–concepts and applications. Int. J. Pharm. Res. Tech..

[62] Bedair T.M., Yu S.J., Im S.G., Park B.J., Joung Y.K., Han D.K. (2015). Effects of interfacial layer wettability and thickness on the coating morphology and sirolimus release for drug-eluting stent. J. Colloid Interface Sci..

[63] Spijker H., Graaff R., Boonstra P., Busscher H., van Oeveren W. (2003). On the influence of flow conditions and wettability on blood material interactions. Biomaterials.

[64] Qi P., Maitz M.F., Huang N. (2013). Surface modification of cardiovascular materials and implants. Surf. Coatings Technol..

[65] Ranade S.V., Miller K.M., Richard R.E., Chan A.K., Allen M.J., Helmus M.N. (2004). Physical characterization of controlled release of paclitaxel from the TAXUS™ Express^2^™ drug-eluting stent. J. Biomed. Mater. Res. Part A.

[66] Shanshan C., Lili T., Yingxue T., Bingchun Z., Ke Y. (2013). Study of drug-eluting coating on metal coronary stent. Mater. Sci. Eng. C.

[67] Hu T., Lin S., Du R., Fu M., Rao Q., Yin T., Huang Y., Wang G. (2017). Design, preparation and performance of a novel drug-eluting stent with multiple layer coatings. Biomater. Sci..

[68] Sevim K., Pan J. (2018). A model for hydrolytic degradation and erosion of biodegradable polymers. Acta Biomater..

[69] Raval A., Parikh J., Engineer C. (2011). Mechanism and in Vitro Release Kinetic Study of Sirolimus from a Biodegradable Polymeric Matrix Coated Cardiovascular Stent. Ind. Eng. Chem. Res..

[70] Zhu X., Braatz R.D. (2015). A mechanistic model for drug release in PLGA biodegradable stent coatings coupled with polymer degradation and erosion. J. Biomed. Mater. Res. Part A.

[71] Vos N.S., Fagel N.D., Amoroso G., Herrman J.-P.R., Patterson M.S., Piers L.H., van der Schaaf R.J., Slagboom T., Vink M.A. (2019). Paclitaxel-coated balloon angioplasty versus drug-eluting stent in acute myocardial infarction: The REVELATION randomized trial. JACC Cardiovasc. Interv..

[72] Wei Z., Wang W., Zhou C., Jin C., Leng X., Li Y., Zhang X., Chen S., Zhang B., Yang K. (2020). In vitro degradation and biocompatibility evaluation of fully biobased thermoplastic elastomers consisting of poly (β-myrcene) and poly (l-lactide) as stent coating. Polym. Degrad. Stab..

[73] El-Hayek G., Bangalore S., Dominguez A.C., Devireddy C., Jaber W., Kumar G., Mavromatis K., Tamis-Holland J., Samady H. (2017). Meta-Analysis of Randomized Clinical Trials Comparing Biodegradable Polymer Drug-Eluting Stent to Second-Generation Durable Polymer Drug-Eluting Stents. JACC Cardiovasc. Interv..

