# Supplementary material for: Heparin-Tagged PLA-PEG Copolymer-Encapsulated Biochanin A-Loaded (Mg/Al) LDH Nanoparticles Recommended for Non-Thrombogenic and Anti-Proliferative Stent Coating
Source: Int J Mol Sci. 2021 May 21;22(11):5433. doi: 10.3390/ijms22115433 (PMC8196732; doi:10.3390/ijms22115433)
Supplement: Supplementary file 1 [file ijms-22-05433-s001.zip › ijms-1139133-supplementary.pdf]

Supporting Information

Heparin tagged PLA-PEG copolymer encapsulated Biochanin-A loaded (Mg/Al) LDH nanoparticles for non-thromogenic and anti-proliferative stent coating

Shivakalyani Adepu[1], Hongrong Luo [2], Seeram Ramakrishna[1*]

[1]Center for Nanofibers and Nanotechnology, National University of Singapore, Singapore 119260 (shivakalyani@iith.ac.in)

[2]Engineering Research Center in Biomaterials, Sichuan University, Chengdu 610064, China; hluo@scu.edu.cn

[1]Center for Nanofibers and Nanotechnology, National University of Singapore, Singapore 119260 (seeram@nus.edu.sg)

1. *Particle Size of Mg/Al (LDH) nanoparticles*

The average hydrodynamic diameter and PDI of LDH nanoparticles were shown in Fig. S1. Hydrodynamic diameter was found to be 241.1 nm. PDI was 0.272 which is <1.Thus it can be said that the nanoparticles were monodispersed.

			Size (d.nm):	% Intensity	Width (d.nm):
Z-Average (d.nm):	241.1	Peak 1:	222.9	100.0	48.37
PdI:	0.272	Peak 2:	0.000	0.0	0.000
Intercept:	0.982	Peak 3:	0.000	0.0	0.000
Result quality :	Good				

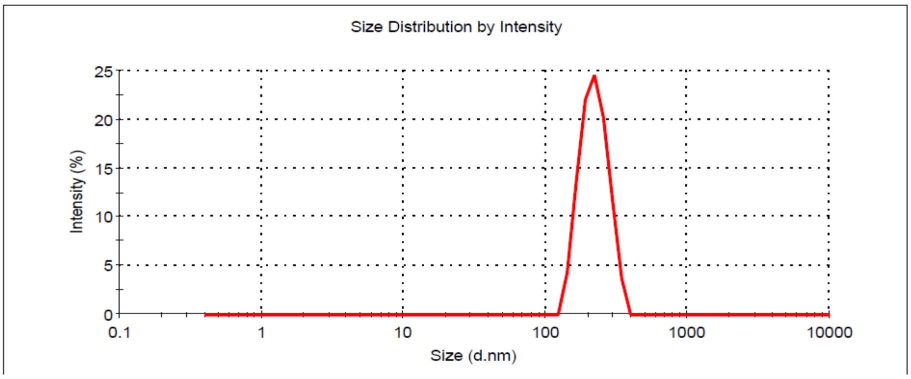

Figure S1 Hydrodynamic diameter and Polydispersity index (PDI) of aqueous suspension of LDH nanoparticles

2. *Zeta potential of Mg/Al(LDH)*

The zeta potential of Mg/Al (LDH) was shown in Fig. S2. It was found to be 26.7mV at pH≈7. At pH lower than the isoelectric point, (pI of LDH ≈11.3), LDH nanoparticles were covered with positively charged stern layer followed by a diffused electrical double layer. The brucite layers in Mg–Al LDH are itself positively charged. At pH 7, the substantially high value of zeta potential 26.7 mV, indicates significant amount of electrical double layer repulsion among the positively charged layers of LDH nuclei.

**Zeta Potential (mV):**	26.7
**Zeta Deviation (mV):**	4.02
**Conductivity (mS/cm):**	0.0823
**Result quality :**	Good

	Mean (mV)	Area (%)	Width (mV)
**Peak 1:**	26.7	100.0	4.02
**Peak 2:**	0.00	0.0	0.00
**Peak 3:**	0.00	0.0	0.00

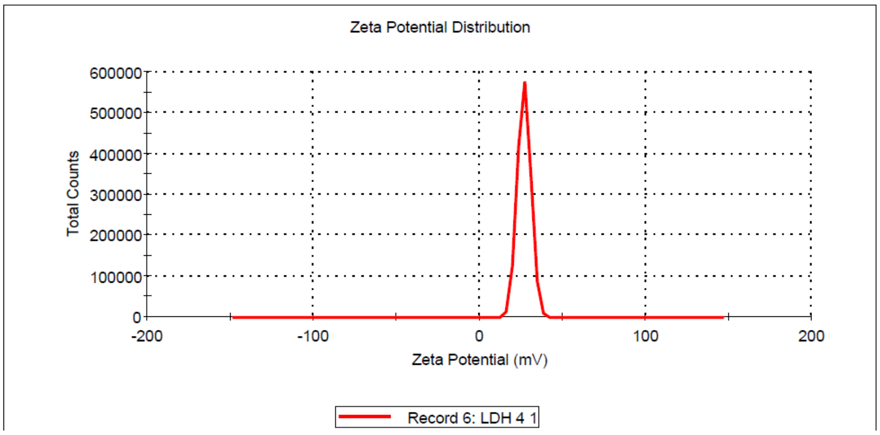

Figure S2 Zeta potential of Mg/Al (LDH)

Zeta potential of BCA-LDH-I and BCA-LDH-C were depicted in fig.S3 & S4, respectively. A decrease in zeta potential from 26.7 (LDH) to 15 (BCA-LDH) was observed, which can be

Results

**Zeta Potential (mV):**	-6.21
**Zeta Deviation (mV):**	4.35
**Conductivity (mS/cm):**	0.00293
**Result quality :**	Good

	Mean (mV)	Area (%)	Width (mV)
**Peak 1:**	-6.21	100.0	4.35
**Peak 2:**	0.00	0.0	0.00
**Peak 3:**	0.00	0.0	0.00

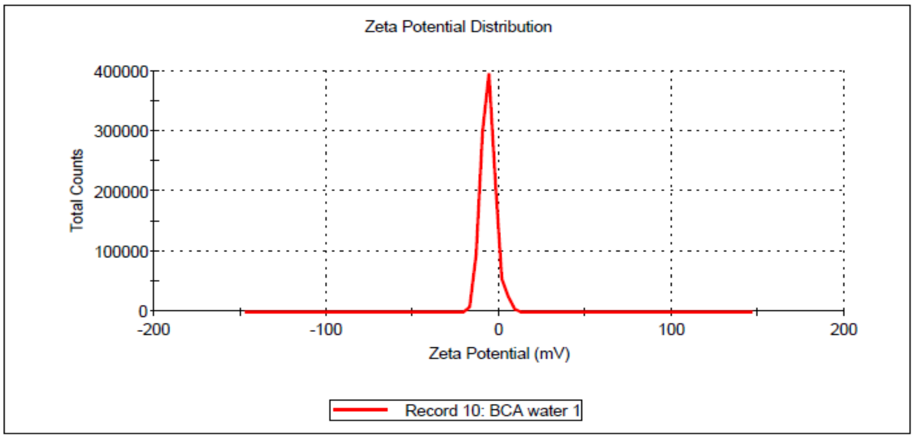

attributed to the high electronegativity of BCA

Figure S3 Zeta potential of Biochanin A

Zeta potential of BCA-LDH-I and BCA-LDH-C shown in fig.S4 and fig. S5, respectively. The zetapotential of BCA-LDH-I was (16.7 mV), BCA-LDH-C (14.7 mV) decreased due to the increase in the surface negative charge after ion exchange with respect to Mg/Al(LDH) (26.7 mV).

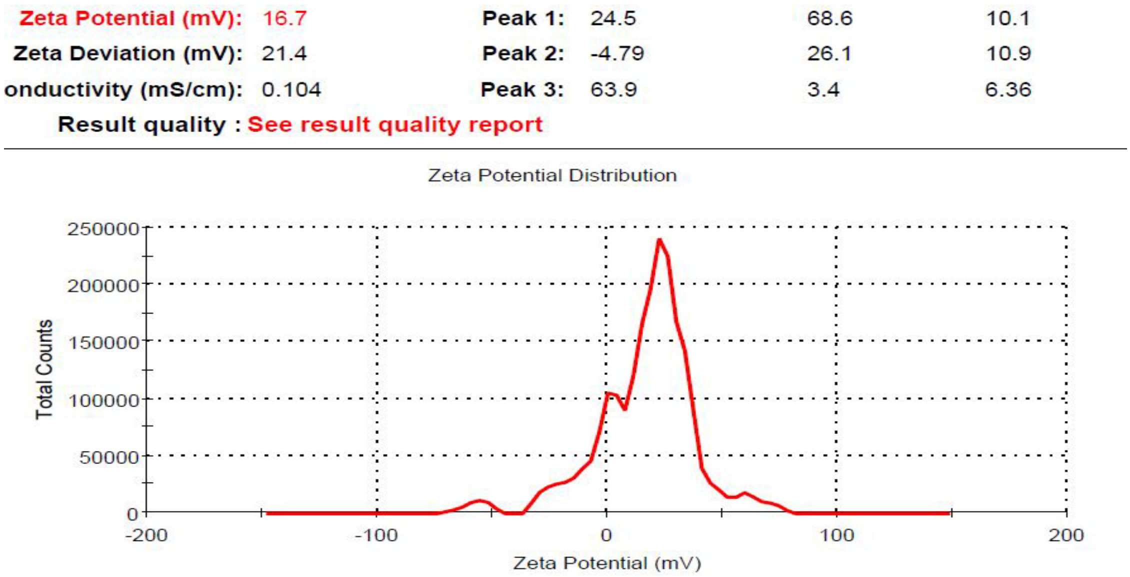

Figure S4 Zeta potential of BCA-LDH-I

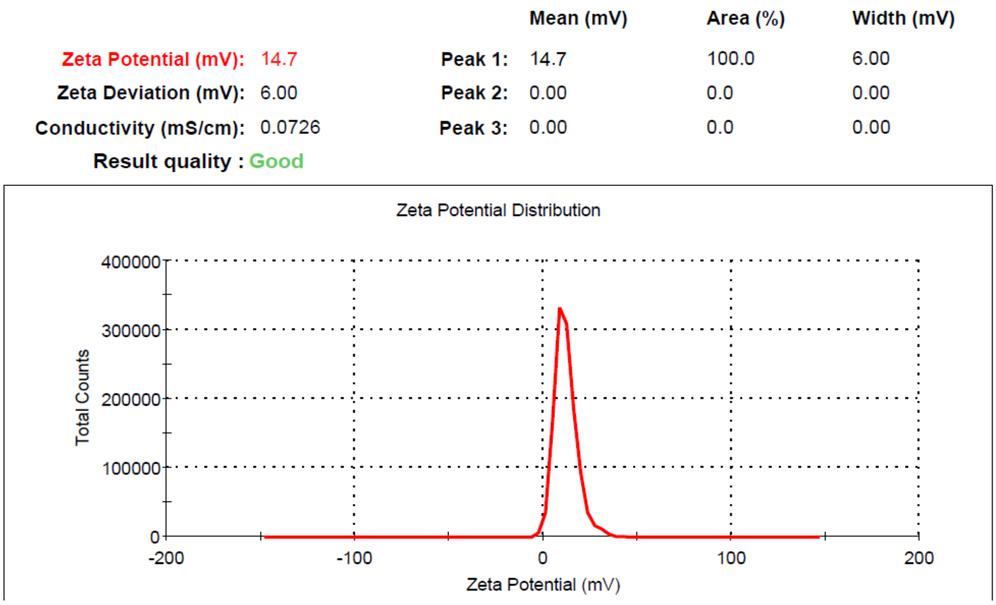

Figure S5 Zeta potential of BCA-LDH-C

3. Analytical method development of BCA and drug loading in LDH nanoparticles

3.1 Absorption maxima of Biochanin A

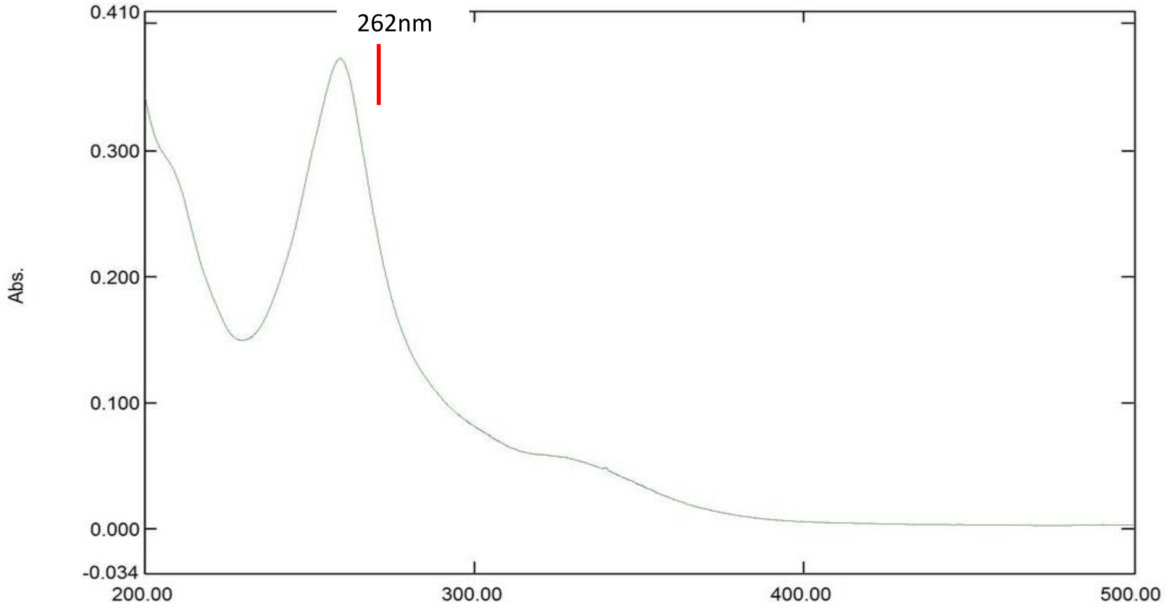

Figure S6 UV-Visible spectrum of Biochanin A

Fig.S6 represents the UV-visible spectra of BCA taken in the scan range of 190-500nm. It shows a strong peak at 262nm and a weak band or shoulder at 300-350nm.

Fig. S7 represents a standard five point calibration curve of BCA from 1 to 5 µg/ml. The R^2 value is pretty closer to 1, indicating the curve is linear.

From Fig.18, the zeta potential of Biochanin A in aqueous suspension was found to be -6.21 mV, which may be attributed to the negative charges on BCA. This negative value depicts the electronegativity of BCA and one can expect better anion exchange with LDH.

3.2 Calibration curve of Biochanin A

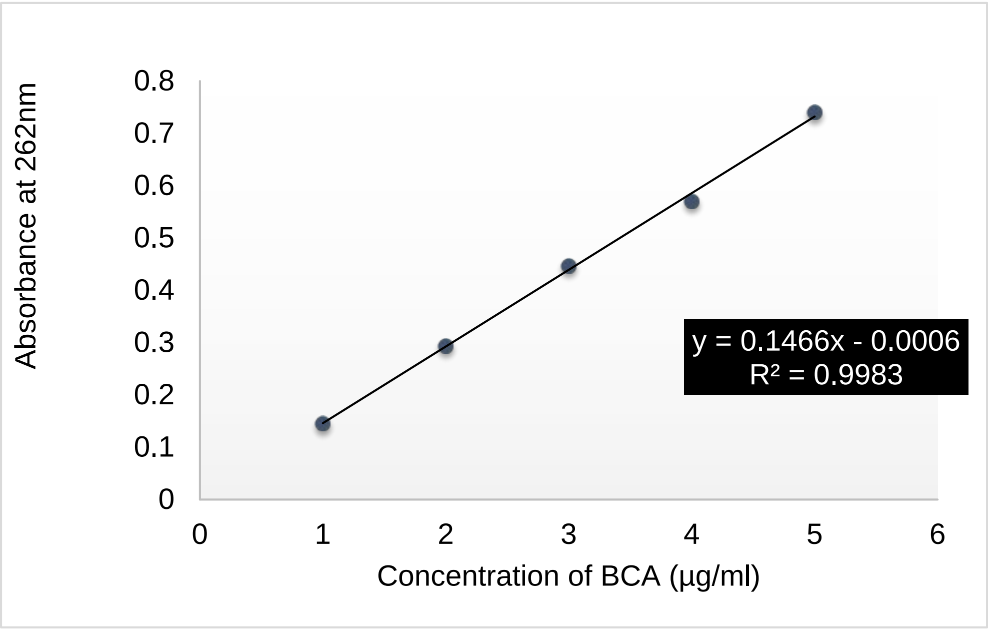

Figure S7 Calibration curve of Biochanin A
